# Delivery of Molecular Hydrogen for Precision Immunomodulation: Mechanisms, Detection Methods, and Applications

**DOI:** 10.1002/advs.202500283

**Published:** 2025-07-06

**Authors:** Gangfeng Li, Hannan Cui, Ruixiao Fan, Guming Liu, Zishuo Hou, Yibo Zhang, Xuanming Che, Tengjiao Wang, Hongbo Wei, Peng Li

**Affiliations:** ^1^ Frontiers Science Center for Flexible Electronics (FSCFE), Institute of Flexible Electronics (IFE) & Institute of Biomedical Materials and Engineering (IBME) Northwestern Polytechnical University (NPU) 127 West Youyi Road Xi'an Shaanxi 710072 China; ^2^ State Key Laboratory of Oral & Maxillofacial Reconstruction and Regeneration, National Clinical Research Center for Oral Diseases, Shaanxi Engineering Research Center for Dental Materials and Advanced Manufacture, Department of Implants, School of Stomatology The Fourth Military Medical University Xi'an Shaanxi 710032 China; ^3^ RWTH Aachen University 52072 Aachen Germany; ^4^ Chongqing Innovation Center Northwestern Polytechnical University Chongqing 401135 China

**Keywords:** gaseous signaling molecule, immune regulation, macromolecular approach, molecular hydrogen, target delivery

## Abstract

The delivery of molecular hydrogen (H_2_) is increasingly recognized for its potential in precision immunomodulation, primarily through multiple pathways such as antioxidant action, modulation of inflammatory factors, and inhibition of apoptosis. These effects contribute to a balanced and functional immune system, highlighting the therapeutic promise of H_2_ in various inflammatory conditions. However, two critical issues remain unresolved: the unclear mechanism underlying the immunomodulatory effects of H_2_ and the lack of precision detection methods to clarify the dose‐efficacy relationship in vivo. Since previous reviews focused on developing nanomaterials for H_2_ delivery, they often overlook analyses of the immunomodulation mechanisms and fail to update the detection methods. To address these gaps, a review that explores the immunomodulatory mechanisms of H_2_ is proposed, the recent detection methods, and the classification and applications of advanced H_2_ delivery systems, aiming to enhance the understanding of precision immunomodulation strategies leveraging their properties.

## Graphical abstract

This review focuses on the immunomodulatory mechanisms of molecular hydrogen, the recent detection methods, and the classification advanced molecular hydrogen delivery materials. It provides a detailed description of their applications in areas such as arthritis, chronic wounds, cardiovascular injury, tumors, and bacterial infections, aiming to enhance the understanding of precision immunomodulation strategies by leveraging the unique properties of molecular hydrogen.

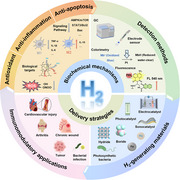

## 1 Introduction

Hydrogen is the simplest and most widely distributed element in nature, and one of the most abundant elements in the human body.^[^ ^1^ ^]^ Recent research has increasingly demonstrated that molecular hydrogen (H_2_), as a novel gaseous signaling molecule, possesses significant immunomodulatory effects, including anti‐apoptotic, anti‐inflammatory, and selective antioxidant responses.^[^ ^2^, ^3^ ^]^ Compared to other “star” gaseous signaling molecules such as nitric oxide (NO), carbon monoxide (CO), and hydrogen sulfide (H_2_S),^[^ ^4^, ^5^, ^6^, ^7^ ^]^ H_2_ is non‐cytotoxic even at high concentrations and exhibits superior biological safety, making it an attractive candidate for therapeutic applications, particularly in the modulation of immune responses.^[^ ^8^, ^9^ ^]^ The therapeutic potential of H_2_ was first noted in 1975 when Dole et al. reported that high‐pressure H_2_ inhibited the growth of mouse skin tumors through its antioxidant properties.^[^ ^10^ ^]^ However, the initial discovery did not receive widespread attention due to the potential safety hazards associated with high‐pressure H_2_, combined with underdeveloped technology. In 1996, David Jones proposed that H_2_ could neutralize hydroxyl radicals (·OH) produced by inflammatory cells attacking pathogens, without causing toxicity to the human body.^[^ ^11^ ^]^ Although initially speculative, this hypothesis was confirmed in 2007, when studies demonstrated that H_2_ selectively scavenged harmful free radicals, thereby reducing inflammation and ischemia‐reperfusion injury.^[^ ^12^ ^]^ Building on these early findings, more recent studies have highlighted the potential of H_2_ in treating various immune‐related diseases, including arthritis, chronic wounds, cardiovascular disease, colon inflammation, and hepatitis.^[^ ^13^ ^]^

Despite the promising findings regarding H_2_’s immunomodulation potential, effective clinical application remains limited due to challenges in its administration. Current methods, such as straightforward H_2_ inhalation, drinking H_2_‐rich water, and injection of H_2_‐saturated saline.^[^ ^14^, ^15^ ^]^ Additionally, H_2_ water baths, gels, and patches have been explored for localized conditions like skin inflammation.^[^ ^16^ ^]^ However, these methods face limitations such as low H_2_ loading capacity, difficulty in achieving controllable delivery to deep tissue lesions, and inability to sustain therapeutic concentrations over time.^[^ ^17^ ^]^ Nanotechnology has revolutionized the field of H_2_ delivery by enabling the development of precise and targeted delivery systems for H_2_ using nanomaterials.^[^ ^18^ ^]^ Since 2017, photon‐driven nanoreactors encapsulating H_2_‐generating catalysts have been used to locally increase H_2_ concentrations, reducing reactive oxygen species (ROS) levels and pro‐inflammatory factors in inflammation‐induced paw edema mice.^[^ ^19^ ^]^ The continuous development of H_2_ delivery nanomaterials has made it possible to precisely control the concentration, duration, and site of action. This ensures sustained therapeutic levels at the target site, minimizes off‐target effects and enhances the overall efficacy of H_2_ delivery in immunomodulation.

**Figure 1:**
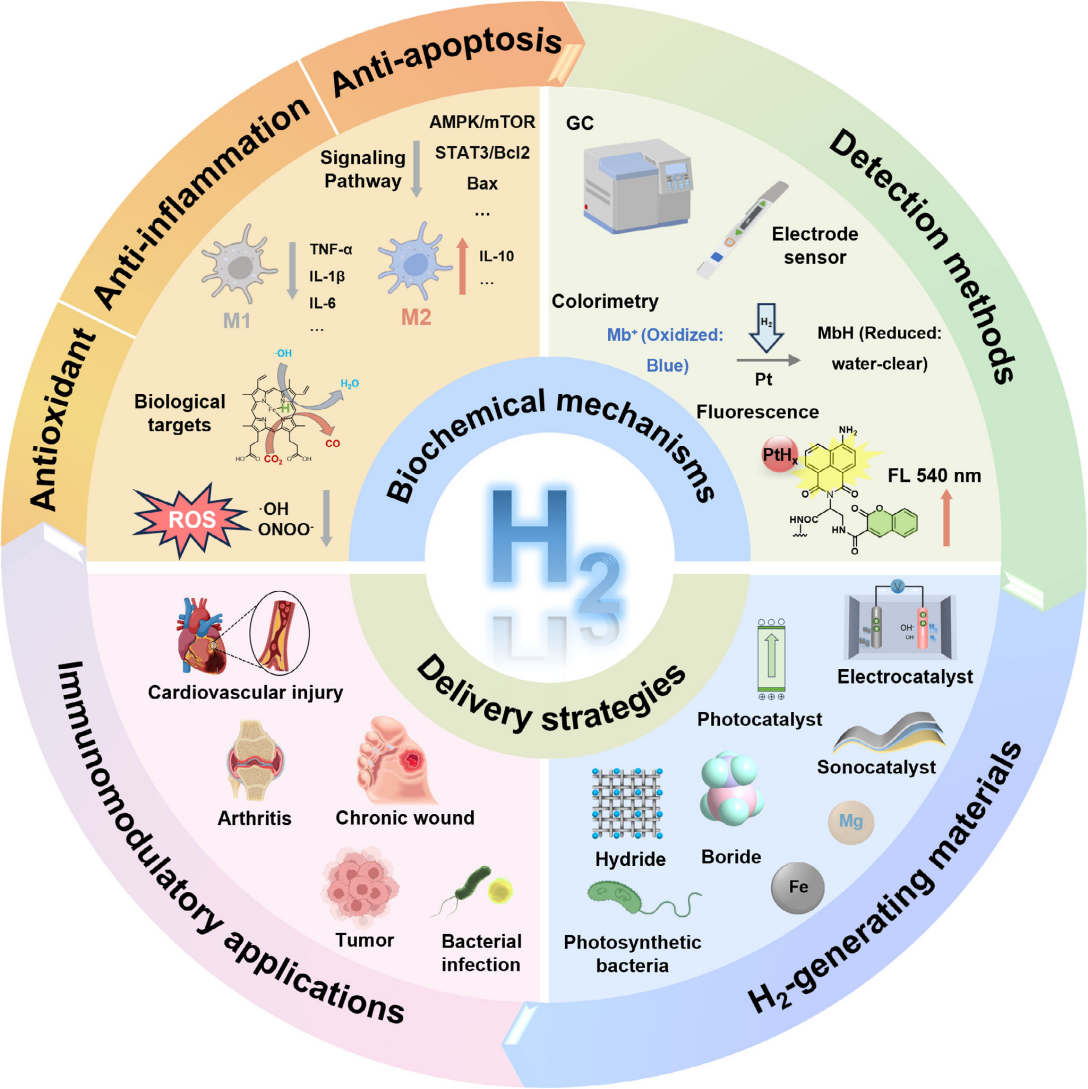
Schematic illustration of H_2_ delivery for immunomodulation.

Although substantial progress has been made, the underlying mechanisms by which H_2_ modulates immune responses remain not fully understood. Moreover, due to the small molecular size, low polarity, and strong tissue penetration ability of H_2_, it can rapidly cross barriers such as the blood‐brain barrier, which are typically impermeable to most drugs.^[^ ^20^, ^21^ ^]^ Therefore, in vivo detection of H_2_ is crucial for understanding its biological effects, in vivo transport, targeting behavior, and the relationship between dosage and efficacy.^[^ ^22^ ^]^ However, existing reviews have primarily focused on the development of materials for H_2_ delivery, often overlooking the analysis of immunomodulatory mechanisms and failing to update detection methods. To address these gaps, this review aims to explore the immunomodulatory mechanisms of H_2_, recent advancements in detection methods, and the classification and applications of advanced H_2_ delivery systems, to enhance the understanding of precision immunomodulation strategies based on H_2_ delivery (Figure 1).

## 2 Mechanisms of H_2_ for Immunomodulation

Inflammation is closely related to oxidative stress, which significantly influences immune responses.^[^ ^23^ ^]^ On the one hand, inflammation can trigger cell aging and release reactive oxygen species (ROS), and excessive ROS causes oxidative stress in the body.^[^ ^24^, ^25^ ^]^ On the other hand, ROS promotes the synthesis of pro‐inflammatory factors such as tumor necrosis factor‐alpha (TNF‐α) by up‐regulating nuclear factor kappa‐B (NF‐κB) signaling pathway.^[^ ^26^ ^]^ These pro‐inflammatory factors activate the expression of triphosphopyridine nucleotide (NADPH) oxygenase, thus promoting NADPH synthesis of ROS, further aggravating the inflammatory response of the body.^[^ ^27^, ^28^ ^]^ H_2_ has been shown to play a key role in mitigating oxidative stress and modulating the immune response.^[^ ^29^ ^]^ In 2007, Ohsawa et al. found that H_2_ can selectively scavenge highly strong oxidants such as ·OH and peroxynitrite (ONOO^−^) in cells and mitigate oxidative damage to proteins, DNA, etc., without affecting other biological ROS.^[^ ^12^ ^]^ Since then, many studies have focused on the immunomodulatory mechanism of H_2_.^[^ ^30^ ^]^ However, it has been shown that the direct reaction of H_2_ with ∙OH and ONOO^−^ is inefficient due to the low water solubility (1.6 ppm) and high activation energy of H_2_.^[^ ^22^ ^]^ Therefore, researchers have hypothesized that enzymes exist in vivo to catalyze reactions involving H_2_. In 2022, *He Qianjun* designed free and protein‐confined Fe‐porphyrin to selectively neutralize highly toxic ∙OH through catalytic hydrogenation, thereby mediating the anti‐oxidation, anti‐inflammation and anti‐aging of H_2_. In the hypoxic microenvironment (such as the tumor hypoxic region), Fe‐porphyrin reduces CO_2_ to CO by catalytic hydrogenation, then mediates the CO signaling pathway in situ, and finally achieves therapeutic effects such as anticancer and immune regulation.^[^ ^31^ ^]^ This study first proposed and confirmed that Fe‐porphyrin serves as a biological target for H_2_, while also elucidating its mitochondrial regulatory effects and systemic impact on immunomodulation.

H_2_ regulates the expression of inflammatory genes through related signal transduction pathways. Numerous studies have shown that H_2_ can reduce inflammation by reducing the release of pro‐inflammatory factors, such as TNF‐α, interleukin‐1β (IL‐1β), interleukin‐6 (IL‐6), interferon‐γ (INF‐γ), intercellular cell adhesion molecule‐1 (ICAM‐1) and high‐mobility group box protein 1 (HMGB‐1), while upregulating the anti‐inflammatory cytokine interleukin‐10 (IL‐10).^[^ ^13^, ^19^, ^32^, ^33^ ^]^ Besides, H_2_ has been shown to mitigate excessive inflammatory responses and reduce endothelial damage in sepsis by activating the Nrf2/HO‐1 signaling pathway.^[^ ^34^ ^]^ Cardinal et al. further reported that H_2_ suppresses the production of pro‐inflammatory factors by inhibiting the mitogen‐activated protein kinase (MAPK) signaling pathway, thereby alleviating the inflammatory response of kidney transplantation.^[^ ^35^ ^]^ Beyond classical models of inflammation, recent findings indicate that H_2_ regulates the immune microenvironment in neurodegenerative contexts. For instance, He et al. showed that hydrogen‐rich water significantly alleviates oxidative stress, reduces neuroinflammatory cytokines (e.g., TNF‐α, IL‐6, IL‐1β), and restores gut microbiota composition in a zebrafish Alzheimer's disease model, thereby highlighting H_2_’s immunomodulatory roles across the gut‐brain axis.^[^ ^36^, ^37^ ^]^

Meanwhile, H_2_’s regulation of autophagy is context dependent, with H_2_ promoting or inhibiting autophagic depending on factors such as tissue type, disease state, and signaling pathway activation. In cardiomyocyte hypertrophy models, H_2_ inhibits excessive autophagy to protect against pathological remodeling.^[^ ^38^ ^]^ Similarly, in lipopolysaccharide‐induced acute lung injury, H_2_‐saturated saline attenuates tissue damage by suppressing overactivated autophagy through the ROS/AMPK/mTOR signaling pathway.^[^ ^39^ ^]^ In contrast, in lung cancer cells, H_2_ enhances both apoptosis and autophagy by inhibiting STAT3/Bcl2 signaling, and interestingly, suppression of autophagy further amplifies the pro‐apoptotic effects of H_2_.^[^ ^40^ ^]^ Moreover, increasing evidence indicates that H_2_ exerts antiapoptotic effects in normal tissues by significantly reducing the expression of apoptosis‐related proteins such as Bax, caspase 3, and caspase 9.^[^ ^41^ ^]^ However, the precise signaling pathways through which H_2_ regulates the expression of these proteins remain unclear. Additionally, it is still uncertain whether H_2_ affects mitochondrial structure and function to interfere with the apoptotic process.

Despite these significant advances, several key questions remain regarding the molecular mechanisms of H_2_‐mediated immunomodulation. For instance, it is still unclear whether H_2_ has additional molecular targets beyond its known effects on oxidative stress and apoptosis‐related proteins, and whether other gaseous signaling molecules such as NO or H_2_S can exert similar downstream effects through catalytic or signaling pathways. Moreover, experimental evidence is needed to clarify whether H_2_ can interfere with Fenton‐like reactions involving Fe/Mn/Zn‐porphyrins, thereby halting the progression of immune‐related diseases.^[^ ^31^ ^]^ In addition, although the four gaseous signaling molecules (H_2_, NO, CO, and H_2_S) are known to modulate immune responses, studies specifically investigating the interactions between H_2_ and the other NO and H_2_S are still very limited. Given their overlapping signaling pathways, such as NF‐κB and MAPK, both synergistic and antagonistic interactions are biologically plausible. For example, NO and H_2_S have been shown to coordinate vascular responses and blood pressure regulation, while preliminary studies have suggested potential interplay between H_2_ and CO.^[^ ^42^ ^]^ In contrast, direct interaction between H_2_ and NO or H_2_S remains largely unexplored. These gaps point to important future research directions for better understanding how gaseous signaling molecules regulate the immune system. Future studies are required to clarify the relative contributions of these mechanisms and to understand better the molecular pathways through which H_2_ mediates its immunomodulatory effects.

## 3 Methods of H_2_ Detection

**Figure 2:**
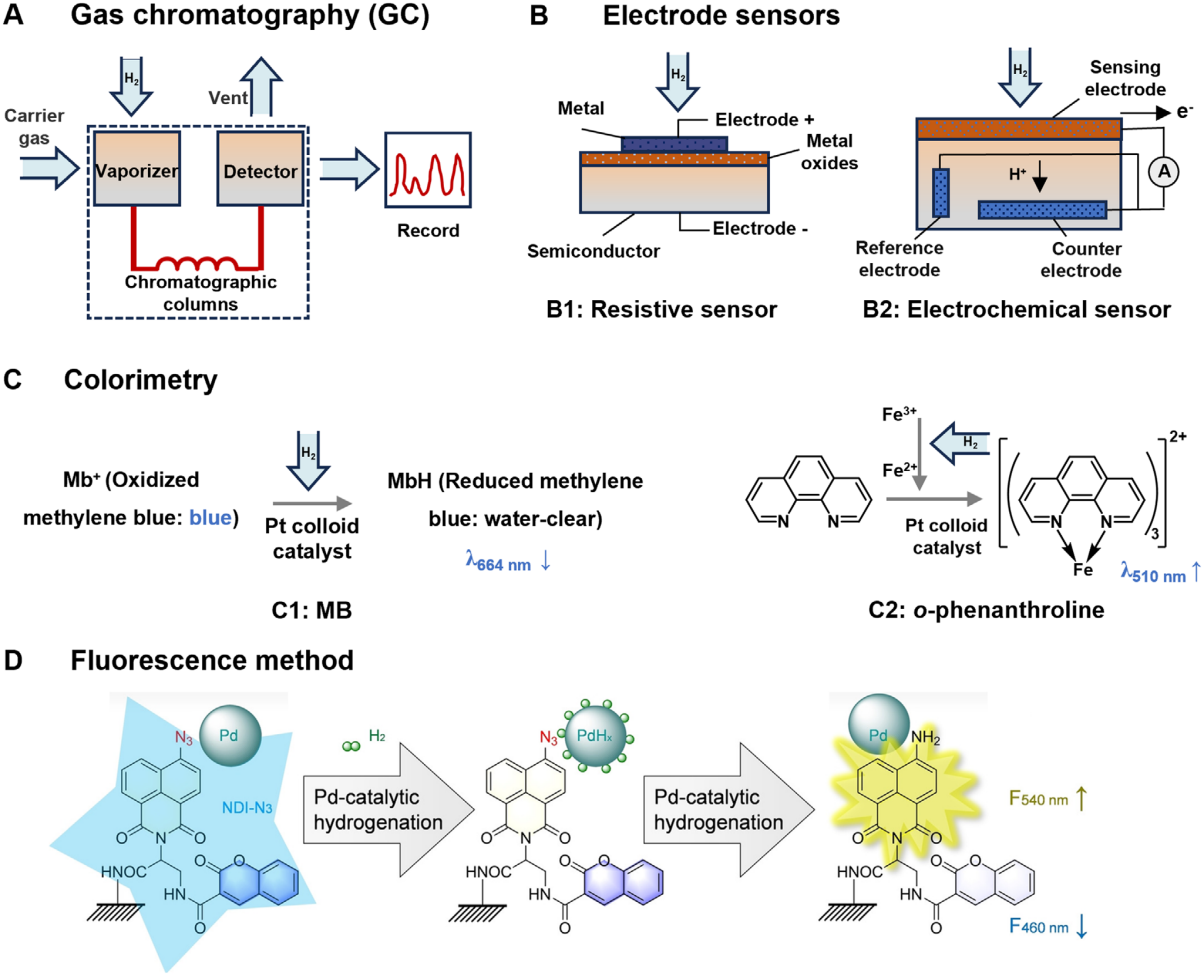
Mechanism of H_2_ detection methods. A) Gas chromatography. B) Electrode sensors (resistive sensor and electrochemical sensor). C) Colorimetry (MB and *o*‐Phenanthroline). D) Fluorescence method. Reproduced with permission.^[^ ^22^ ^]^ Copyright 2022, Wiley‐VCH.

H_2_ concentration detection is essential for studying the distribution, in vivo dynamics, and dose‐effect relationships of H_2_ in the body. In the early stages of the H_2_ study, gas chromatography (GC) became the mainstream method for detecting H_2_ concentrations in solutions or blood due to its high precision and accuracy, and has since been widely adopted in biomedical studies. For instance, the first seminal paper on H_2_ biology published in 2007 employed this method, and subsequent studies have used GC to monitor changes in blood H_2_ levels following H_2_ inhalation (**Figure** **2**A).^[^ ^12^, ^43^ ^]^ Additionally, mass spectrometry (MS) is capable of detecting extremely low concentrations of H_2_, however, its application in H_2_ biomedical research remains limited. Notably, a gas chromatography‐mass spectrometry (GC‐MS) method with selected ion monitoring (SIM) (GC‐MS/SIM) offers a viable approach for the quantitative determination of H_2_ in biological samples without necessitating complex pretreatment. In this approach, helium is used as the carrier gas to separate both the analyte and the internal standard. A calibration curve for H_2_ quantification in the gas phase is established based on the peak area ratio of H_2_ to ^22^Ne. This GC‐SIM‐MS method exhibited high sensitivity, with a detection limit of 1.7 ppm for H_2_ analysis.^[^ ^44^ ^]^ However, GC and MS are limited by complex equipment, bulky size, strict requirements on environmental conditions, and high cost, which limits the practical application of these methods. Consequently, electrode sensing methods with stronger portability and real‐time detection capabilities, are increasingly favored.^[^ ^45^, ^46^ ^]^ Among them, the resistive semiconductor sensor achieves effective detection of H_2_ concentration through the electrochemical analysis principle of gas adsorption materials.^[^ ^47^, ^48^ ^]^ Sang Hun Kim et al. developed a uniquely structured H_2_ sensor by combining hierarchical porous SnO_2_ nanostructures and PdO, which exhibits excellent selectivity, response speed, sensing kinetics and a short recovery period. Notably, the sensor achieved a low detection limit of 0.096 ppm for H_2_ in exhaled breath.^[^ ^49^ ^]^ Research based on CuO sensors has further refined the detection performance of H_2_ through the design of composite materials. For H_2_ detection in solution, electrochemical H_2_ sensors are widely used in many fields due to their fast and accurate response characteristics. This type of sensor absorbs and decomposes H_2_ through the sensing electrode, and then quantifies the H_2_ concentration through current detection (Figure 2B). This method is not only used to monitor the biodegradation process but also to evaluate the impact of H_2_ concentration on osteogenesis‐related cell types during the degradation of magnesium alloys in vivo. In addition, H_2_ detection devices incorporating electrochemical principles, such as carbon‐based microelectrodes modified with [NiFe] hydrogenase, broaden the applicability of H_2_ detection.

The colorimetry based on redox reactions offers an economical and practical approach for detecting H_2_ content in aqueous solutions. This method involves the redox reaction between methylene blue (MB) and H_2_ under the catalysis of colloidal platinum. The procedure consists of adding MB solution dropwise until the solution turns blue, indicating the presence of H_2_. Notably, the colorless solution will turn blue upon exposure to oxygen in the air, complicating the direct spectrophotometric detection of H_2_ in water, as the solution's color changes due to the interaction with atmospheric oxygen. This method enables detection of H_2_ concentrations approximately between 0.2 and 1.6 mg L^−1^.^[^ ^50^ ^]^ Additionally, a simple and rapid UV‐vis spectroscopic method for determining the concentration of H_2_ in aqueous solutions under acidic conditions is based on the redox reaction between iron ions and H_2_ in the presence of colloidal platinum. The released ferrous ions are then quantified by colorimetric reaction with *o*‐phenanthroline, and the absorbance is measured at 510 nm (Figure 2C). This method achieves a detection limit of 0.696 µM for H_2_ in water.^[^ ^51^ ^]^ However, it should be noted that this type of method may be interfered with by other oxidizing or reducing substances in the solution, limiting its application in complex samples such as blood and cell culture fluid.

Molecular imaging probes enable real‐time tracking of H_2_ within the body, offering greater temporal and spatial flexibility while minimizing tissue damage.^[^ ^52^ ^]^ In addition, fluorescence spectroscopy with molecular imaging probes provides a new perspective on detecting H_2_ in vivo. A ratio‐type fluorescence H_2_ probe was designed and synthesized by conjugating Pd nanoparticles and azide/coumarin‐modified fluorophores to PEG‐modified mesoporous silica nanoparticles (NDI‐N_3_/Pd@MSN‐PEG). The probe utilizes the hydrogenation catalysis of small Pd nanoparticles encapsulated in the mesoporous silica, which rapidly captures H_2_ and catalyzes their conversion to highly reactive hydrogen atoms. This process transforms NDI‐N_3_ into NDI‐NH_2_, resulting in fluorescence emission at 540 nm (Figure 2D). The developed H_2_ probe exhibits high selectivity, rapid response, high sensitivity, and low detection limits (26.7 nM).^[^ ^22^ ^]^ The probe has been successfully used to observe the ultrafast transmembrane transport of H_2_ in cells, animals, and plants, showing great potential as a universal tool for studying the role of H_2_ in immunomodulation.

## 4 H_2_ Delivery Materials Categorized by Different H_2_‐Generating Mechanisms

Classifying H_2_ delivery materials based on their H_2_ generation mechanisms has become an essential strategy for exploring the effective utilization of H_2_ in immunomodulation, as well as for developing advanced H_2_ delivery systems. In this section, we categorized the H_2_ delivery materials reported for immunomodulation over the past five years into three main types: catalytic materials for H_2_ generation, H_2_ storage materials, and living biomaterials for H_2_ generation (**Table** **1**). This classification offers a comprehensive overview of each category, emphasizing their potential to address the low‐efficiency limitations of traditional methods.

**Table 1:** H_2_ delivery materials categorized by different H_2_ generation methods in the past five years.

Classification	H_2_ generation mechanism	Material	Disease model	H_2_ detection method	Refs.
Catalytic materials for H_2_ generation	Photocatalyst	TiO_2_	Diabetic wound	Electrode	[53]
			OA	Electrode	[54]
			Diabetic wound	GC	[55]
			Pressure ulcer	GC	[56]
		HA‐Au/Ag@ZnS	RA	MB	[57]
		SnS_1.68_‐WO_2.41_	Tumor (4T1 cells and HeLa cells)	Electrode	[58]
		ZrTc‐Co	Tumor (H22 cells)	MB	[59]
		[FeFe]TPP/GEM/FCS	Bladder Cancer (T24 cells)	GC	[60]
		UCNPs@Zn–Co	Tumor (4T1 cells)	GC	[61]
		C_3_N_4_	Tumor (4T1 cells)	MB	[62]
		Pdots	Oxidative stress	GC	[63]
	Electrocatalyst	Fe needle electrode	Tumor (MCF‐7 cells)	n.a.	[64, 65]
		Zn‐Fe primary battery	Type 2 diabetes	GC	[66]
		MgG	Tumor (CT26 cells)	GC	[67]
		MnNi_2_S_3_	Tumor (CT26 cells)	GC	[68]
	Sonocatalyst	ZnS	Deep tumor (Hepa1‐6‐Luc cells)	GC	[69]
		SnS	Deep tumor (Hepa1‐6‐Luc cells)	GC	[70]
		H_2_‐PFOB	Myocardial ischemia	GC	[71]
		Pt‐Bi_2_S_3_	Tumor (4T1 cells)	MB	[72]
H_2_ storage materials	Reductive metal	Fe	Tumor (4T1 cells)	GC	[73]
		Mg	RA	MB	[74]
			Tumor (4T1 cells and MC38 cells)	Electrode	[75]
			Sepsis	MB	[76]
			Deeply burned skin	Electrode	[77]
			Non‐alcoholic fatty liver disease	n.a.	[78]
	Hydride	PdH	Chronic liver diseases	MB	[79]
			Atherosclerosis	MB	[80, 81]
			Melanoma	MB	[82]
		CaH_2_	Liver cancer	MB	[83]
		H‐Si	Oxidative stress	GC	[84]
			OA	MB	[85]
		H_x_WO_3_	Diabetic wound	MB	[86]
	Boride	NH_3_·BH_3_	Tumor (4T1 cells)	MB	[87]
			OA	Sensor	[88]
		CaB_6_	OA	GC	[89]
		MgB_2_	Gastric Cancer (BGC‐823 cells)	MB	[90]
Living biomaterials for H_2_ generation	*Chlorella vulgaris*	Bacillus–*Chlorella*	Diabetic wound	GC	[91]
	Bacteria	Photosynthetic bacteria	Tumor (MCF‐7 cells)	MB	[92]
		*Enterobacter.Aerogenes*	Psoriasis	Electrode	[93]

### 4.1 Catalytic Materials for H_2_ Generation

The concept of catalysis originated in the field of chemistry and has been extensively applied in industries and energy sectors. In recent years, it has progressively transitioned into the biomedical field.^[^ ^94^, ^95^ ^]^ Unlike the high catalytic efficiency pursued in the chemical industry, biomedical research requires catalytic materials with sensitive responsiveness and controlled release.^[^ ^96^ ^]^ A particularly promising application of catalytic materials in biomedicine is H_2_ generation. Based on the energy driving the process, catalytic H_2_ generation can be broadly classified into three distinct categories: photocatalytic, electrocatalytic and sonocatalytic H_2_ generation. These methods employ different forms of energy to induce the catalytic reactions required to produce H_2_, holding the promise of long‐term immunomodulation applications for chronic inflammatory diseases.

#### 4.1.1 Photocatalytic H_2_ Generation

Light‐triggered photocatalytic H_2_ generation offers a highly manipulable approach for on‐demand hydrogen release from water. This process relies on photocatalysts that absorb light and use the energy to drive chemical reactions. In biomedicine, this technique shows significant potential by providing a continuous and controlled release of H_2_, essential for therapeutic applications. Since the discovery of photocatalytic water splitting on TiO_2_ electrodes by Fujishima et al. in 1972, semiconductor photocatalysis has become a key area of research, particularly in the biomedical field.^[^ ^97^ ^]^ This process begins with the absorption of light by semiconductor materials, which excites electrons from the valence band to the conduction band, leaving behind vacancies or holes in the valence band. These separated electrons and holes then drive redox reactions with water molecules: the electrons reduce water to generate H_2_, while the holes oxidize water to generate oxygen.^[^ ^98^ ^]^ This controlled process offers precise spatial and temporal H_2_ release, making it highly promising for targeted therapeutic applications. Among the various semiconductor materials, titanium dioxide (TiO_2_) nanoparticles have garnered particular attention due to their biocompatibility, chemical stability, and non‐toxicity. Recent advancements include the development of near‐infrared (NIR) photocatalysts, such as TiO_2_ nanorods doped with hydrogen atoms have shown enhanced efficiency under visible and NIR light, broadening their applicability in biomedical settings.^[^ ^53^, ^54^ ^]^ To further enhance photocatalytic performance, heterojunction engineering has emerged as a promising strategy. For example, a Bismuth (Bi) nanocrystal‐decorated Bi_2_WO_6_/H‐TiO_2_ heterojunction was constructed to enable dual photocatalytic functionality: glucose degradation and H_2_ generation. Under light irradiation, glucose serves as a sacrificial agent, facilitating effective charge separation and continuous H_2_ production. This rational design not only improves the efficiency of H_2_ generation but also demonstrates the versatility of heterojunction‐based photocatalysts in physiological environments.^[^ ^55^ ^]^ In addition, hydrogen‐doped TiO_2_ nanorods decorated with palladium nanodots (HTON‐Pd) have recently been developed as a multifunctional photocatalyst capable of simultaneously producing H_2_ and mild thermal energy under visible light irradiation. This dual‐functional design offers a promising platform for the development of light‐responsive materials with precise spatiotemporal control and holds great potential for integration into advanced biomedical H_2_ generation materials.^[^ ^56^ ^]^

However, despite the advances in NIR‐responsive photocatalysts, tissue‐specific light penetration remains a critical constraint for in vivo applications. NIR light exhibits good penetration in superficial tissues such as skin or subcutaneous tumors, making it well‐suited for treating shallow lesions. In contrast, its penetration in deep tissues like the brain is limited, often necessitating the use of high‐intensity light sources or invasive optical fibers. These practical considerations underscore the importance of aligning light‐responsive catalyst design with the anatomical characteristics of the target site to maximize therapeutic outcomes while minimizing potential tissue damage. To address limitations such as charge recombination in narrow‐bandgap materials, Z‐type nanocatalysts like SnS_1.68_‐WO_2.41_, which combine high reduction and oxidation potentials, have been developed to enhance charge separation and NIR absorption, enabling efficient H_2_ generation.^[^ ^58^ ^]^ Building upon this progress, further advancements have led to the development of multiphoton‐responsive NIR photocatalysts. A notable example is ZrTc‐Co, which is based on a Zr‐MOF structure incorporating thiazolothiazole‐based organic ligands (Tc) as model multiphoton‐active units. Surface modification of this material introduces cobalt ions as the photocatalytic active site. ZrTc‐Co is capable of absorbing multiple photons, enabling it to reach higher energy states and significantly enhance photocatalytic hydrogen generation. This material improves electron‐hole separation and optimizes adsorption conditions, making it a promising candidate for deep‐tissue H_2_ delivery.^[^ ^59^ ^]^

Simultaneously, natural and synthetic photosensitizers have further propelled the development of photocatalytic systems. Modified porphyrins, like those used in the [FeFe]TPP/GEM/FCS nanoparticle H_2_ generator, also show significant promise in medical applications, enabling H_2_ generation under specific light conditions.^[^ ^60^ ^]^ Furthermore, metal‐organic frameworks (MOFs) and polymeric semiconductors, such as g‐C_3_N_4_, have been explored to enhance photocatalytic performance by precise tuning and doping.^[^ ^62^ ^]^ These hybrid systems, including semiconductor polymer dots (Pdots), offer tunable optical band gaps and improved photocatalytic activity. For example, researchers have demonstrated the therapeutic potential of Pdots for in situ H_2_ generation using liposomes as nanoreactors.^[^ ^63^ ^]^ Collectively, these advancements in photosensitizers and hybrid systems complement semiconductor‐based photocatalysis, offering new and innovative solutions for controlled H_2_ release in biomedical applications.

#### 4.1.2 Electrocatalytic H_2_ Generation

Electrocatalytic H_2_ generation utilizes electrical energy to generate H_2_ from water, offering precise control over the release of H_2_.^[^ ^99^ ^]^ Traditional Chinese acupuncture needles (electrodes) have provided a novel approach for in vivo electrochemical H_2_ generation. Qi et al. demonstrated that these electrodes facilitate effective electrochemical reactions in acidic environments by applying a low voltage of ≈3 V, leading to localized H_2_ generation.^[^ ^64^, ^65^ ^]^ However, the need for an external power source may make it less practical for future clinical applications. Implantable electrodes allow for precise modulation of H_2_ generation and are particularly promising in neural applications; however, the long‐term biocompatibility and mechanical stability of electrode materials must be carefully considered to ensure safe and sustained use in vivo. Additionally, Zn‐Fe micro/nano‐structures have been engineered to enhance the hydrolysis rate of zinc. By adjusting the Zn‐to‐Fe ratio, these structures enable controlled H_2_ release, providing a sustained and efficient method for H_2_ generation.^[^ ^66^ ^]^ However, the low electrocatalytic activity and instability of Fe needles limit their practical applications.

Magnesium (Mg) and its alloys, widely used in clinical implants, have also been explored as potential materials for localized H_2_ release. While Mg reacts with water to generate H_2_, the slow reaction rate limits its efficiency. To address this, a micro‐galvanic cell was developed by in situ reduction of Pt on Mg rods (MgG). This Mg‐based galvanic cell can spontaneously generate H_2_ without requiring an external power supply, providing a practical approach for localized H_2_ delivery. In vivo studies in mice showed that MgG rods implanted in 4T1 tumors degraded efficiently within 15 days, with no significant accumulation of Mg^2+^ in blood or major organs. Pt nanoparticles were gradually cleared, primarily via renal excretion. No histological damage or abnormal liver/kidney function was observed, indicating good biocompatibility and biosafety of both Mg^2+^ and Pt components.^[^ ^67^ ^]^ These findings support the potential of MgG as a safe and biodegradable material for in vivo H_2_ generation. In parallel, recently studies have introduced manganese‐doped Ni_2_S_3_ nanoelectrodes (MnNi_2_S_3_ NEs) as an innovative electrocatalyst for enhancing electrocatalytic H_2_ generation. These nanoelectrodes significantly improve the H_2_ evolution reaction by reducing the initial energy barrier and promoting the efficient desorption of H_2_, resulting in a more controllable and effective H_2_ generation.^[^ ^68^ ^]^

#### 4.1.3 Sonocatalytic H_2_ Generation

Sonocatalytic H_2_ generation utilizes ultrasonic energy to drive water‐splitting reactions, presenting a promising method for generating H_2_ in various therapeutic contexts. Ultrasound offers excellent tissue penetration and is especially applicable to deep‐seated tissues such as joints and the brain; however, excessive energy input may result in thermal or mechanical damage, making precise control of ultrasound parameters essential for safe application. Recent developments in this field have introduced sonocatalysts that enhance efficiency and control over H_2_ generation. For instance, mesocrystalline ZnS nanoparticles have been engineered to achieve effective sonocatalytic full water splitting, providing a sustained release of hydrogen and oxygen.^[^ ^69^ ^]^ In addition, 2D SnS nanosheets have emerged as an effective sonocatalyst, leveraging their strong piezoelectric effect for ultrasound‐driven H_2_ generation. These SnS nanosheets enable efficient H_2_ generation at a low power density of ultrasound, making them highly suitable for localized and deep tissue applications.^[^ ^70^ ^]^ Building on this progress, perfluorinated compounds (PFCs) have emerged as a complementary technology for enhancing hydrogen generation in biomedical applications. PFCs are inert organic compounds with a high affinity for oxygen, making them particularly useful for improving tissue oxygenation. Due to their excellent biocompatibility, PFCs are widely used in clinical applications such as artificial blood substitution, organ preservation, ultrasound imaging, and ^19^F magnetic resonance imaging. Among them, H_2_‐loaded PFOB (perfluorooctylbromide) nanodroplets stand out for their high H_2_ storage capacity. These PFOB‐based nanoemulsions exhibit large solubilization capacity for oxygen, facilitated by van der Waals interactions, and can trigger explosive hydrogen release when subjected to low‐intensity focused ultrasound (LIFU). The low‐frequency, low‐intensity characteristics of LIFU offer the added benefit of an excellent biosafety profile, allowing precise and controlled H_2_ delivery to targeted sites.^[^ ^71^ ^]^ These advances underscore the potential of sonocatalytic materials for precise and efficient H_2_ generation across a range of biomedical applications. Additionally, bismuth sulfide (Bi_2_S_3_) combined with in situ Pt nanoparticles has demonstrated improved catalytic performance by optimizing charge separation and H_2_ generation, even under hypoxic conditions.^[^ ^72^ ^]^

### 4.2 H_2_ Storage Materials

H_2_ storage materials can generate H_2_ without external power sources or catalysts. These materials can be divided into three main categories based on their composition: reductive metals, hydrides, and borides.

#### 4.2.1 Reductive Metals

Reductive metals, such as iron (Fe) and Mg, are highly sensitive to acids and can generate H_2_, making them beneficial for targeted H_2_ delivery. However, they are also unstable under physiological conditions. Fe nanoparticles coated with carboxymethyl cellulose (CMC) have been developed to address this problem. The CMC coating protects Fe from oxidation, and the small‐sized Fe@CMC nanoparticles exhibit excellent passive targeting properties as well as high responsiveness to H_2_ generation in the presence of weak acids.^[^ ^73^ ^]^ Expanding on the potential of Mg and Fe, these metals are not only used as biodegradable materials in medical devices but also show promise as H_2_‐generating agents. For instance, Sung proposed a poly(lactic acid)‐hydroxyacetic acid copolymer (PLGA) microparticle system containing Mg powder (Mg@PLGA MPs).^[^ ^100^ ^]^ The hydrophobic PLGA hinders water penetration into Mg@PLGA MPs, thus limiting the release rate of H_2_. Additionally, Li et al. developed a biocompatible Mg micromotor coated with hyaluronic acid (HA). The H_2_ bubbles generated by Mg serve as propellants, enabling the micromotor to be transported to the targeted site for treatment, demonstrating a novel approach to H_2_ delivery.^[^ ^74^ ^]^ In addition, the CaCO_3_ nanoparticles‐coated magnesium system (Mg‐CaCO_3_),^[^ ^75^ ^]^ biodegradable tobramycin‐loaded magnesium micromotor (Mg‐Tob motor),^[^ ^76^ ^]^ and 2D Mg_2_Si nanosheet (MSN)^[^ ^77^, ^78^ ^]^ all leverage Mg‐based systems for efficient H_2_ generation, highlighting the unique properties of Mg as a biodegradable, reactive metal that enables H_2_ delivery in various applications.

#### 4.2.2 Hydrides

Palladium(Pd)‐based hydrides (PdH) are recognized for remarkable H_2_ storage capabilities due to their unique interaction with hydrogen atoms. It enables targeted H_2_ delivery and controlled release, particularly when exposed to near‐infrared (NIR) light. In particular, the unique spike‐like morphology of PdH nanoparticles further enhances H_2_ release and facilitates interactions with surrounding tissues, making them promising for biomedical applications.^[^ ^79^, ^80^, ^81^, ^82^ ^]^ Building on the foundation of PdH, metal hydrides like calcium hydride (CaH_2_) and magnesium hydride (MgH_2_) serve as potent spontaneous H_2_ generate materials. For instance, CaH_2_ can continuously generate H_2_ through a reaction with water:

(1) $$\mathrm{Ca}H_{2}+2H_{2}O=\mathrm{Ca}{\left(\mathrm{OH}\right)}_{2}+2H_{2}$$

This characteristic is further enhanced when nano‐CaH_2_ is dispersed in low molecular weight polyethylene glycol (PEG), leading to the generation of abundant H_2_ upon contact with water.^[^ ^83^ ^]^

Unlike metal hydroxides, which are by‐products of H_2_ generation from alkali metals and may complicate treatment processes, H‐Si reacts rapidly and continuously with ambient water to generate H_2_ without requiring external energy input. This presents a more efficient and sustainable method for H_2_ generation, highlighting the continuous advancements in hydride‐based H_2_ delivery technology.^[^ ^84^, ^85^ ^]^ However, one challenge in developing in situ H_2_ generation systems is the need for reactions with water and acids to trigger H_2_ release. An important but often overlooked aspect of these materials is their biocompatibility, which is critical for clinic applications. To address this, there is a growing interest in finding alternative carriers that can directly store high‐reduction potential hydrogen atoms. A promising candidate material is tungsten bronze (H_x_WO_3_, 0 < x < 1), a solid‐state H_2_ carrier where hydrogen atoms can be reversibly inserted and extracted. The biocompatibility of tungsten‐oxide materials has been widely validated, which reduces the risk of physiological rejection and post‐treatment toxicity, making them an attractive option for safe and effective H_2_ delivery.^[^ ^86^ ^]^

#### 4.2.3 Borides

Boride‐based materials, including ammonia borane (AB, NH_3_·BH_3_),^[^ ^87^ ^]^ calcium hexaboride (CaB_6_),^[^ ^89^ ^]^ and magnesium boride (MgB_2_),^[^ ^90^ ^]^ are emerging as promising candidates for spontaneous H_2_ generation, offering an efficient, catalyst‐free method for H_2_ release under specific conditions. NH_3_·BH_3_, with its high hydrogen content and impressive volumetric hydrogen density of 152.9 g L^−1^,^[^ ^101^ ^]^ undergoes a hydrolysis reaction in acidic conditions to release H_2_, as represented by the reaction:

(2) $$NH_{3}\cdot BH_{3}+H^{+}+3H_{2}O=N{H_{4}}^{+}+B{\left(\mathrm{OH}\right)}_{3}+3H_{2}$$

NH_3_·BH_3_ remains stable in neutral and alkaline environments but releases H_2_ rapidly under acidic conditions, with the process accelerated by heat. When embedded in polymer matrices or porous microspheres, AB provides controlled, sustained H_2_ release, making it suitable for targeted biomedical applications.^[^ ^88^ ^]^ Similarly, CaB_6_ and MgB_2_ react with water to produce H_2_ and metal hydroxides, particularly under acidic conditions. These boride‐based materials offer an effective means for spontaneous H_2_ generation, which can be harnessed for controlled H_2_ delivery.

### 4.3 Living Biomaterials for H_2_ Generation

The above traditional H_2_ generation methods are often limited by reaction conditions, the need for catalysts, and material consumption. Once the materials are depleted, H_2_ generation cannot be sustained, and residual byproducts may be generated during the reaction, posing potential safety risks in medical applications. Biological H_2_ generation leverages the natural metabolic pathways of microorganisms to generate H_2_, offering a sustainable and eco‐friendly approach to H_2_ generation. Wu et al. developed a symbiotic algae‐bacterial gel patch incorporating Chlorella and Bacillus licheniformis, where the respiration of Bacillus licheniformis establishes an oxygen‐deprived environment crucial for activating hydrogenase in *Chlorella*, leading to H_2_ generation during photosynthesis.^[^ ^91^ ^]^ Both green algae and photosynthetic bacteria (PSB) are known to generate H_2_ under light and anaerobic conditions. Green algae primarily use water as a substrate, while PSB utilizes organic compounds, showcasing the diverse biological pathways available for H_2_ generation in these microorganisms.^[^ ^92^ ^]^ Additionally, Zhang's group encapsulated H_2_ generation *Enterobacter aerogenes* within microneedles, maintaining bacterial viability while continuously generating H_2_ through its metabolic processes. This approach enables precise control over the depth and rate of drug release. Collectively, these approaches highlight the potential of biological systems inefficient and targeted H_2_ generation for various applications.^[^ ^93^ ^]^

## 5 Precision Immunomodulation Applications of H_2_ Delivery Materials

### 5.1 ROS Scavenging in Arthritis

Osteoarthritis (OA) and rheumatoid arthritis (RA) are complex autoimmune and inflammatory diseases that lead to severe damage to joint tissues, resulting in high morbidity and mortality.^[^ ^102^ ^]^ The excessive generation of ROS and pro‐inflammatory cytokines such as IL‐1β, IL‐6, and TNF‐α play critical roles in the pathogenesis and progression of OA and RA. ROS can directly damage chondrocytes, synovial cells, and osteocytes, leading to cell apoptosis and death.^[^ ^103^ ^]^ Additionally, ROS activate inflammatory signaling pathways, promoting the release of inflammatory mediators and exacerbating arthritis. Eliminating ROS with H_2_ to reduce inflammation has emerged as a novel and promising approach for treating arthritis, but its rapid diffusion and short retention time pose challenges to achieving stable therapeutic effects.^[^ ^84^, ^85^, ^104^ ^]^

**Figure 3:**
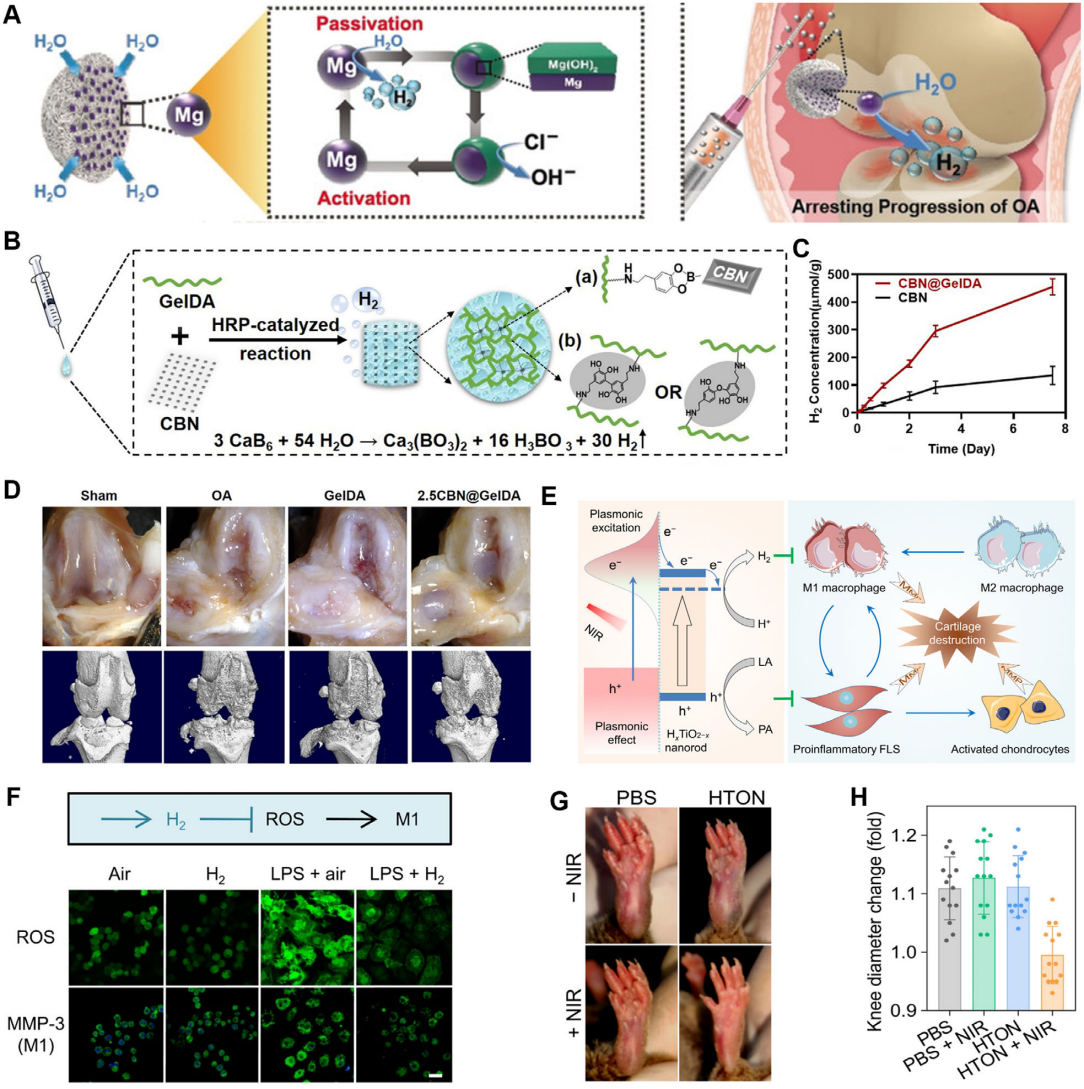
A) Schematic illustration of H_2_ generation from Mg@PLGA NPs arresting the progression of OA. Reproduced with permission.^[^ ^100^ ^]^ Copyright 2018, Wiley‐VCH. B) Schematic illustration of the fabrication process of the CBN@GelDA hydrogel. C) The cumulative H_2_ release behaviors of CBN@GelDA hydrogel in PBS solutions at 37 °C. D) Representative photographs and micro‐CT scanning of the rat knee joint on day 28 after treatment with different groups. Reproduced with permission.^[^ ^89^ ^]^ Copyright 2023, Elsevier. E) Schematic illustration of NIR‐photocatalytic strategy and mechanism for arthritic SME regulation with the NIR‐responsive HTON. F) The effects of H_2_ on intracellular ROS level and macrophage polarization. Scale bar: 20 µm. G) The representative digital pictures of left lower paws of RA mice after 39‐day treatment. H) Knee diameter change of RA mice after 32‐day treatment. Reproduced with permission.^[^ ^54^ ^]^ Copyright 2022, American Association for the Advancement of Science.

To enable effective immunomodulation utilizing H_2_ in inflamed joints, various intelligent H_2_ delivery systems have been developed, particularly those utilizing Mg as a core material due to its biodegradability and ability to generate H_2_ in situ. Wan et al. designed Mg@PLGA MPs for localized and controlled H_2_ release in OA treatment (**Figure** **3**A). Upon intramuscular injection near the affected joints, these microparticles gradually generate H_2_ through the controlled degradation of Mg, effectively scavenging ROS and reducing inflammation without causing local alkalization.^[^ ^100^ ^]^ This approach successfully mitigated tissue inflammation and prevented cartilage destruction, highlighting its potential for clinical application. Similarly, Xu et al. developed biocompatible Mg‐based micromotors coated with hyaluronic acid for precise RA management. These micromotors, administered intra‐articularly under ultrasound guidance, continuously produce H_2_ that not only propels the micromotors for enhanced tissue penetration but also effectively scavenges ROS and downregulates pro‐inflammatory cytokines. This strategy significantly alleviated joint damage and suppressed arthritis severity in a collagen‐induced arthritis rat model, demonstrating enhanced therapeutic efficacy through targeted and sustained H_2_ delivery.^[^ ^74^ ^]^

Direct liquid injection of nanocarriers for H_2_ delivery can lead to the easy loss of the nanocarriers from the injection site, potentially causing joint cartilage abrasion.^[^ ^105^ ^]^ To address this issue, hydrogels, as commonly used drug and nanoparticle delivery systems, have emerged as a promising solution. Hydrogels can reduce joint damage by minimizing physical friction and ensuring controlled, localized release of therapeutic agents.^[^ ^106^, ^107^ ^]^ Zhang et al. formulated an injectable hydrogel composed of CaB_6_ nanosheets embedded in dopamine‐grafted gelatin (CBN@GelDA hydrogel) as a high‐payload and sustainable H_2_‐releasing system for OA treatment (Figure 3B).^[^ ^89^ ^]^ The hydrogel provides a stable and biocompatible matrix that facilitates continuous H_2_ release over an extended period, effectively reducing oxidative stress and inflammatory responses while promoting the polarization of macrophages toward the anti‐inflammatory M2 phenotype (Figure 3C). A single injection of CBN@GelDA hydrogel markedly reduced joint destruction and improved cartilage integrity in OA rat models, offering a promising strategy for the long‐term management of inflammatory joint diseases (Figure 3D). In addition, stem cell‐based strategies have recently incorporated H_2_ release mechanisms to further improve therapeutic outcomes in OA. For instance, Zhou et al. engineered H_2_ generators‐backpacked mesenchymal stem cells, which home to OA joints after systemic administration and generate H_2_ in response to the acidic microenvironment. The released H_2_ mitigates mitochondrial dysfunction, reduces apoptosis, maintains paracrine activity, and reverses redox and immune dysregulation. Notably, this approach also promotes M2‐type macrophage polarization and enhances cartilage regeneration, highlighting the synergistic benefits of combining cellular therapy with local H_2_ delivery.^[^ ^88^ ^]^

Despite these advances, on‐demand and sustained H_2_ release remains crucial for enhancing precision immunomodulation in arthritis. To address this need, researchers have developed additional strategies for controlled H_2_ delivery. For example, electrospinning technology has been used to encapsulate H‐Si nanosheets into gelatin methacryloyl short fibers, enabling slow, long‐term H_2_ release in rat bone joints. This approach aims to reduce the reaction rate of H‐Si and achieve sustained therapeutic effects.^[^ ^85^ ^]^ Building on these strategies, He et al. developed highly stable, monodisperse H_2_‐doped TiO_2_ (H_x_TiO_2‐x_) nanorods (HTON) for NIR photocatalytic modulation of the RA synovial microenvironment (SME) (Figure 3E).^[^ ^54^ ^]^ Under NIR light irradiation, HTON not only generates H_2_ locally to scavenge ROS in pro‐inflammatory M1 macrophages, inhibiting their pro‐inflammatory phenotype and preventing cartilage damage, but also consumes excess lactate (LA) generated in the SME, preventing its invasion into cartilage and thus correcting the SME (Figure 3F,G). Furthermore, a significant reduction in knee joint diameter was observed in RA mice (Figure 3H). This dual‐function strategy provides an effective avenue for precision immunomodulation in arthritis.

### 5.2 Chronic Wound Healing

Chronic wounds, such as diabetic foot ulcers (DFUs) and severe burns, are characterized by persistent inflammation, oxidative stress, and microbial infection.^[^ ^108^, ^109^ ^]^ In DFUs, high glucose levels contribute to the accumulation of advanced glycation end products, leading to excessive ROS generation, chronic inflammation, and impaired tissue regeneration. Conventional drugs for chronic wound treatment often face secondary failure, leading to suboptimal outcomes.^[^ ^110^ ^]^ H_2_ is capable of activating various endogenous stem cells and promoting the production of autologous collagen, thereby inducing a new wound‐healing pattern and accelerating the healing process.^[^ ^111^ ^]^

**Figure 4:**
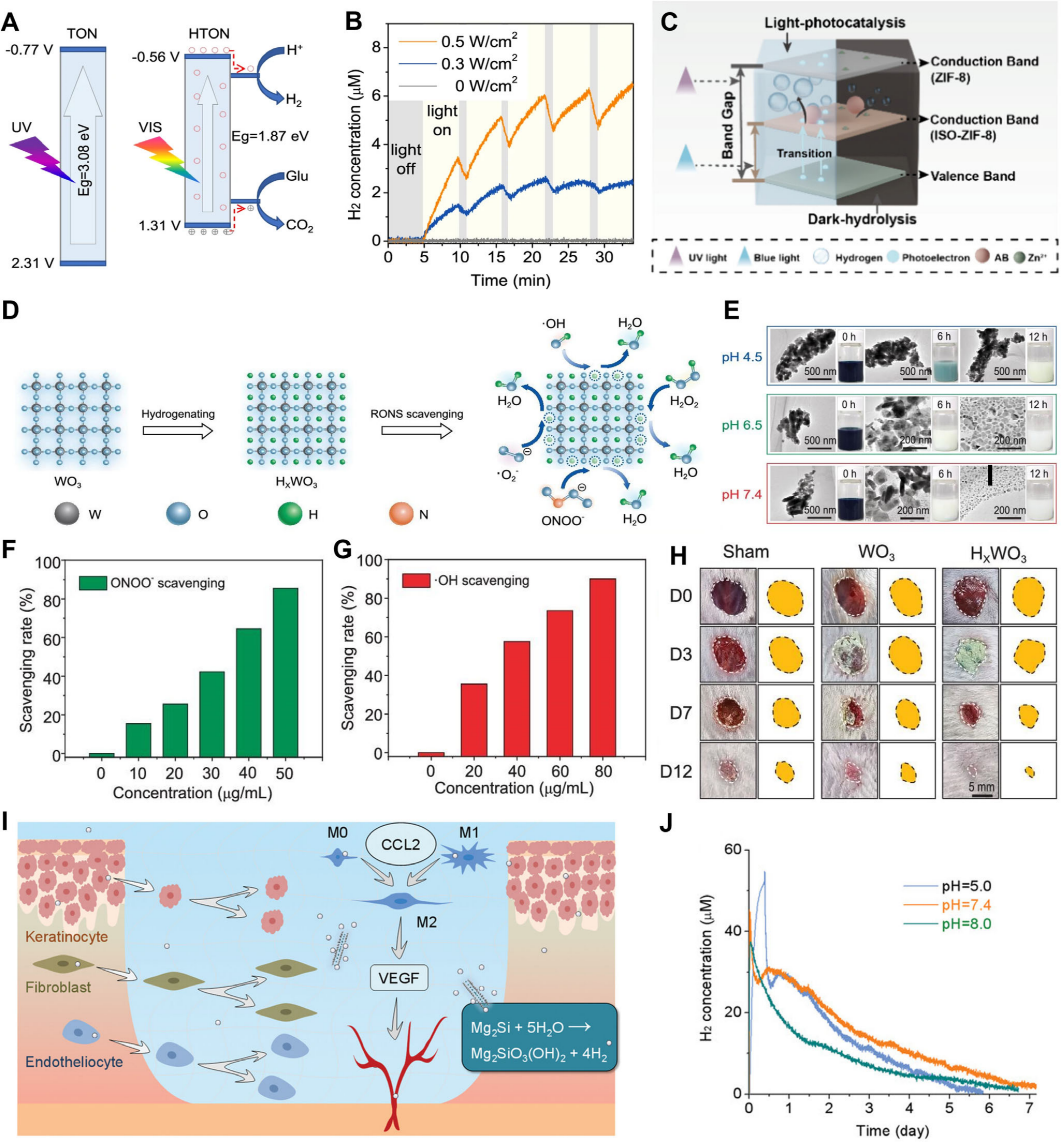
A) Band structures of TON and HTON and the schematic illustration of the mechanism for VIS‐photocatalytic H_2_ generation and glucose consumption. B) VIS‐photocatalytic H_2_ generation behavior of the HTON nanocatalyst in the aqueous solution of glucose (20 mM) under irradiation of xenon lamp at various power densities. Reproduced with permission.^[^ ^53^ ^]^ Copyright 2022, Springer Nature. C) Schematic illustration of H_2_ release from the ISO‐ZIF‐8@AB nanoplatforms. Reproduced with permission.^[^ ^112^ ^]^ Copyright 2025, American Chemical Society. D) Schematic diagram of preparation of H_x_WO_3_ and RONS scavenging. E) HRTEM images displaying the collapse of H_x_WO_3_ NPs during the pH‐responsive degradation from 0 to 12 h. The scavenging rate of F) peroxynitrite anion (ONOO^−^) and G) hydroxyl radicals (·OH) at different H_x_WO_3_ concentrations. H) Representative images of the dynamic wound healing process on day 0, 3, 7, and 12 treated with PBS, WO_3_ and H_x_WO_3_. Reproduced with permission.^[^ ^86^ ^]^ Copyright 2024, China Science Publishing & Media Ltd. I) Wound healing strategy and mechanisms of sustained release of hydrogen from the MSN@CS/HA dressing. J) H_2_ release profiles of the MSN@CS/HA hydrogel dressing in the PBS solutions with different pH values. Reproduced with permission.^[^ ^77^ ^]^ Copyright 2023, Wiley‐VCH.

Recent advances in H_2_ delivery materials have facilitated the use of H_2_ in the setting of chronic wound infection. Chen et al. developed an H_2_‐delivery hydrogel dressing made from live *Bacillus* and *Chlorella vulgaris*, which generates H_2_ under light activation to accelerate diabetic wound healing.^[^ ^91^ ^]^ The high‐glucose environment in diabetic wounds often limits the effectiveness of immunomodulation. He et al. developed a glucose‐depleting, H_2_‐producing system by combining a titanium oxide nanorod (HTON) with an appropriate bandgap structure, acting as a visible (VIS)‐light‐sensitive photocatalyst. This system utilizes glucose as a sacrificial agent to generate H_2_ via vis‐emitting xenon lamp photocatalysis (**Figure** **4**A).^[^ ^53^ ^]^ Both H_2_ generation and glucose consumption can be easily controlled by adjusting the irradiation time and power density of visible light, which is beneficial to glucose consumption and immune regulation in HTON‐covered diabetic wounds (Figure 4B). To further enhance spatiotemporal control over H_2_ generate, ISO‐ZIF‐8@AB nanoplatforms were recently developed by modifying ZIF‐8 with isocyanate groups and loading AB. This system combines photocatalytic and hydrolytic mechanisms, enabling visible‐light‐triggered H_2_ generation via bandgap narrowing and Zn^2+^‐assisted AB hydrolysis even in dark conditions (Figure 4C). It achieves a dual‐phase H_2_ release profile, characterized by an initial burst followed by sustained release for over 14 days, thereby better matching the dynamic requirements of inflammation management in chronic wounds.^[^ ^112^ ^]^

While materials like HTON and ISO‐ZIF‐8@AB offer light‐responsive features, other systems bypass external energy input. Zhu et al. proposed a more versatile solution by designing hydrogen tungsten bronze (H_x_WO_3_), which leverages the unique temperature and pH conditions of the diabetic wound environment for atomic hydrogen generation (Figure 4D).^[^ ^86^ ^]^ The material exhibits temperature‐dependent atomic hydrogen release behavior and its unique pH‐responsive biodegradability ensures post‐therapeutic clearance at pathological sites (Figure 4E). Atomic hydrogen produced by H_x_WO_3_ can efficiently convert reactive oxygen and nitrogen species (RONS) into water, modulating the expression of beneficial anti‐inflammatory cytokines (Figure 4F,G). This process stimulates cell proliferation and angiogenesis, ultimately accelerating chronic diabetic wound healing (Figure 4H).

Similarly, severe burn wounds suffer from extensive tissue damage, ischemic reperfusion injury, and oxidative stress, which present a major challenge in wound healing.^[^ ^113^, ^114^ ^]^ To address this, the development of long‐duration H_2_ delivery systems can improve wound healing outcomes. He et al. developed an H_2_‐generating platform using 2D Mg_2_Si nanosheets (MSN), which were incorporated into a chitosan/hyaluronate hydrogel (MSN@CS/HA) for deep burn wound treatment (Figure 4I).^[^ ^77^ ^]^ Compared to previous H_2_ delivery materials, the MSN@CS/HA hydrogel can sustain H_2_ release for up to one week, significantly improving cellular viability, reducing oxidative stress, and promoting angiogenesis and tissue regeneration (Figure 4J). Moreover, the designed MSN@CS/HA hydrogel demonstrates excellent biocompatibility with its degradation products, including H_2_, Mg^2+^, SiO_3_^2−^, CS, and HA, being low in toxicity and easily metabolized in the body. The biocompatibility and extended H_2_ release capability of the MSN@CS/HA hydrogel make it a promising option for burn wound care, offering a durable and safe alternative for H_2_ delivery in immunomodulation.

### 5.3 Cardiovascular Injury Alleviation

H_2_ has been shown to protect against cardiovascular diseases (CVDs) such as ischemia‐reperfusion (I/R) injury, atherosclerosis (AS), myocardial infarction, cardiac remodeling and radiation‐induced cardiac damage.^[^ ^115^, ^116^ ^]^ H_2_ is capable of inhibiting ·OH and ONOO^−^ within cells and tissues, significantly reducing oxidative stress, which in turn leads to a decrease in the hallmark inflammation observed in CVD pathogenesis. Damage to myocardial cells and vascular cells, including endothelial and smooth muscle cells, is a key risk factor for cardiovascular dysfunction in CVDs.^[^ ^117^ ^]^ Additionally, increased fibrosis and apoptosis are closely related to heart failure. Studies have shown that H_2_ treatment (inhaling 2% H_2_ for 3 h daily over 28 days) significantly improved cardiac function in mice with myocardial infarction, reduced fibrotic areas, alleviated hypoxia‐induced cardiac cell injury, and inhibited angiotensin II‐induced migration and activation of cardiac fibroblasts.^[^ ^118^ ^]^

**Figure 5:**
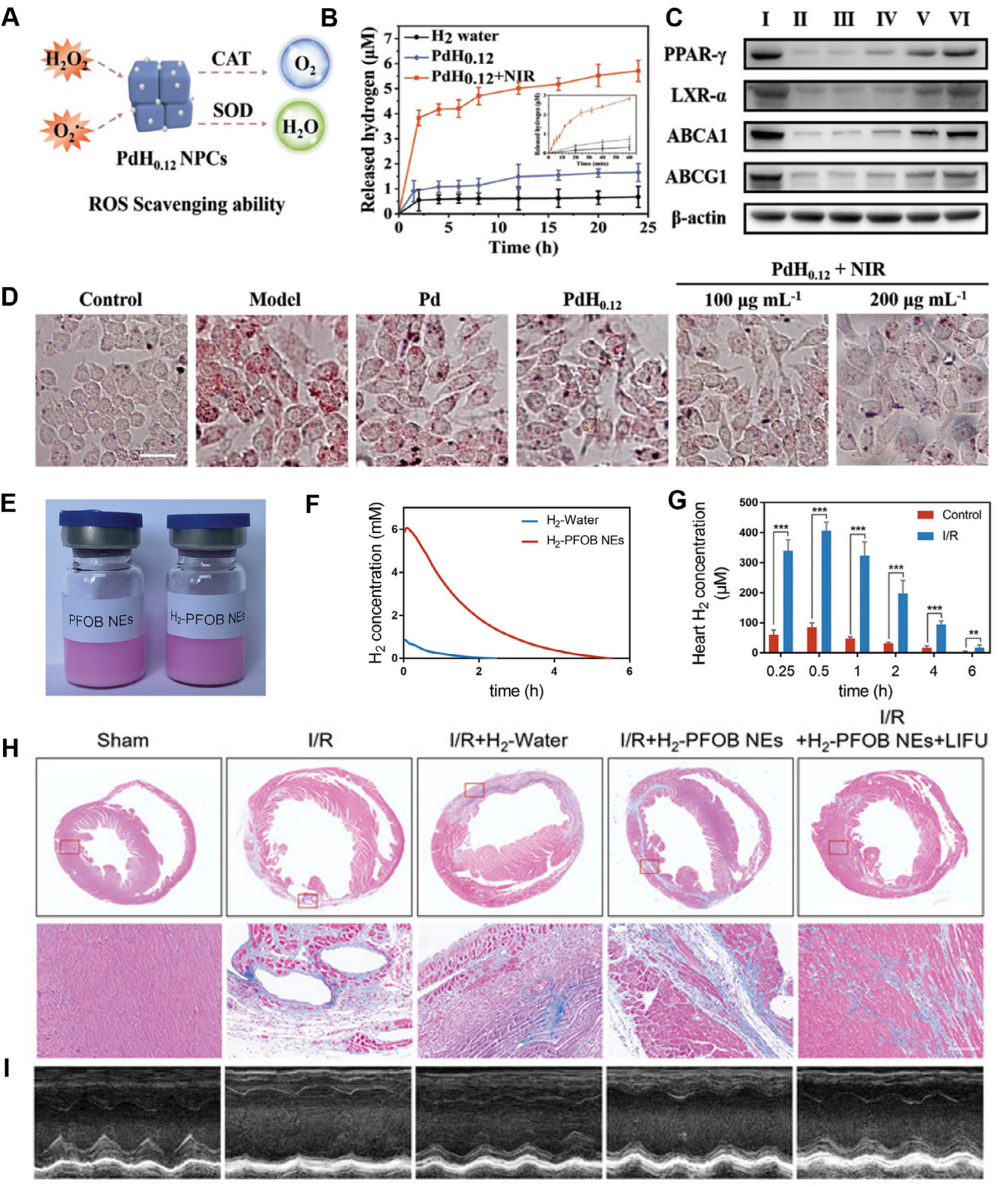
A) Schematic illustration of ROS scavenging ability of PdH_0.12_ NPCs. B) The H_2_ release behavior of H_2_ water, Pd NPCs, and PdH_0.12_ NPCs, as measured using an MB probe. C) Western blotting analysis of PPAR‐γ, LXR‐α, ABCA1, and ABCG1 protein levels in RAW264.7 cells after different treatments. D) Optical microscopy images of oxLDL‐induced foam cell formation in RAW264.7 cells after different treatments. Scale bar: 25 µm. Reproduced with permission.^[^ ^80^ ^]^ Copyright 2021, Wiley‐VCH. E) Representative image of the H_2_‐PFOB NEs. F) Residual H_2_ concentration of H_2_‐Water and H_2_‐PFOB NEs with increasing time. G) H_2_ concentration in normal mice and I/R mice were measured by H_2_ microelectrodes at different times after injection of H_2_‐FPOB NEs. H) The representative Masson images of mice's hearts. I) Representative echocardiography images of mice's hearts. Reproduced with permission.^[^ ^71^ ^]^ Copyright 2023, Wiley‐VCH.

Recently, some research groups have focused on H_2_ delivery to achieve precision immunomodulation in CVDs. Xu et al. developed PdH nanopocket cubes (PdH_0.12_ NPCs) through a seed‐mediated method, employing a “coupling hardness with softness” strategy to amplify the inhibition of foam cell formation and block the progression of AS. In the “hardness” aspect, Pd NPCs act as antioxidant enzymes (catalase and superoxide dismutase) to scavenge ROS, preventing the generation of oxidized low‐density lipoprotein (oxLDL) and weakening lipid uptake (**Figure** **5**A).^[^ ^80^ ^]^ In the “softness” aspect, under NIR‐II laser irradiation, the local temperature rise disrupts the Pd‐H bond, triggering the release of H_2_ (Figure 5B). This leads to the activation of the LXR‐α/ABCA1/ABCG1‐mediated cholesterol transport pathway through upregulation of PPAR‐γ expression, promoting lipid efflux (Figure 5C). The simultaneous reduction in lipid uptake and enhancement of lipid efflux through this “coupling hardness with softness” strategy effectively amplifies the inhibition of foam cells and alleviates the progression of AS lesions (Figure 5D). Similarly, Hu et al. designed PdHs nanoenzymes for targeted AS management. In their approach, Fe species were added during the synthesis to fabricate a distinctive tetrapod needle‐like structure in PdH‐based metal nanoenzymes, endowing them with enhanced autophagic ability. After being internalized by macrophages, TN‐PdHs nanoenzymes accumulate at the atherosclerotic lesions, where their strong ROS‐scavenging capacity, H_2_‐based anti‐inflammatory effects, and autophagy induction work synergistically to exert powerful anti‐atherosclerotic effects. This dual mechanism enhances the anti‐inflammatory and antioxidative properties of the TN‐PdHs, making them a promising approach for AS management.^[^ ^81^ ^]^

However, these PdH nanomaterials have inorganic biosafety issues, and their application in clinical transformation is still challenging. To address these challenges, Nie et al. reported a novel therapeutic approach for myocardial I/R injury using H_2_‐perfluorooctylbromide nanoemulsions (H_2_‐PFOB NEs).^[^ ^71^ ^]^ Building on previous research showing the excellent oxygen solubilization capacity of PFOB NEs through van der Waals interactions, they first demonstrated that these emulsions could also effectively load H_2_ (Figure 5E). Moreover, the release of H_2_ from H_2_‐PFOB NEs could be precisely triggered by LIFU at the target site, leveraging the unique low‐intensity and low‐frequency properties of ultrasound to ensure excellent biosafety. As shown in Figure 5F, compared to H_2_ water, the H_2_ release rate from H_2_‐PFOB NEs is slower, making it suitable for sustained hydrogen release. Furthermore, microelectrode measurements of H_2_ concentration in the hearts of I/R mice revealed that H_2_‐PFOB NEs delivered a higher peak H_2_ concentration and sustained this release for a longer period, confirming their targeted delivery capability to I/R‐injured myocardium (Figure 5G). Additionally, echocardiography results showed that both the H_2_‐PFOB and H_2_‐PFOB+LIFU groups exhibited a significant reduction in myocardial fibrosis area and improvement in cardiac function compared to the H_2_‐water group (Figure 5H,I). This highlights that the combined use of H_2_‐PFOB NEs and LIFU maximizes the protective effects against myocardial I/R injury.

### 5.4 Tumor Microenvironment Modulation

Chronic inflammation plays a pivotal role in cancer progression by releasing cytokines, growth factors, and chemokines, which promote cell proliferation and inhibit apoptosis, thereby creating a microenvironment conducive to tumor development.^[^ ^119^, ^120^ ^]^ H_2_ has been identified as a promising anticancer agent because it inhibits cancer cell energy metabolism, suppresses vascular endothelial growth factor expression, and activates systemic immune responses.^[^ ^121^, ^122^ ^]^ Furthermore, H_2_ exhibits selective antioxidant properties, which play a significant role in preventing tumor initiation.^[^ ^12^ ^]^ Notably, studies have shown that H_2_‐rich electrolyzed water can induce apoptosis in breast cancer cells by reducing the expression of epidermal growth factor receptor 2, and disrupting the phosphorylation of key signaling proteins such as extracellular signal‐regulated kinase and protein kinase B.^[^ ^123^ ^]^ By selectively scavenging ROS, H_2_ alleviates chromosomal damage and inhibits the abnormal activation of tumor‐related signaling pathways. This action facilitates the timely repair of chromosomal damage, thus preventing the initiation and progression of tumors.^[^ ^124^ ^]^ Given that high levels of ROS in tumor tissues contribute to tumor proliferation, migration, and invasion, immune modulation of tumor microenvironment with H_2_ delivery offers a promising strategy for inhibiting tumor growth and metastasis.^[^ ^125^ ^]^

To generate abundant H_2_ in tumor regions, Liu et al. dispersed CaH_2_ nanoparticles in low molecular weight polyethylene glycol (PEG) and injected them into tumors. The nanoparticles react with water to produce H_2_, which modulates the tumor microenvironment (TME).^[^ ^83^ ^]^ Cheng et al. developed a Mg‐based electrochemical battery (MgG) by decorating platinum on the surface of Mg rods. When implanted into tumors, the MgG rods generate H_2_ within the tumor, inducing mitochondrial dysfunction and disrupting the intracellular redox balance. The residual Mg(OH)_2_ can neutralize the acidic TME, inhibiting tumor growth.^[^ ^67^ ^]^ However, these methods do not allow precise control of the H_2_ release time and dosage. Jin et al. developed a clinically available acupuncture needle as electrodes, enabling the controlled in situ generation of sufficient H_2_ in the TME through mild electrochemical reactions. Notably, since H_2_ is also involved in various physiological activities of tumor cells, its release disrupts the delicate balance of the TME, leading to tumor dysfunction. After treatment, the reduced cancer‐associated inflammation facilitates immune system recovery, thereby enhancing therapeutic efficacy.^[^ ^64^, ^65^ ^]^

**Figure 6:**
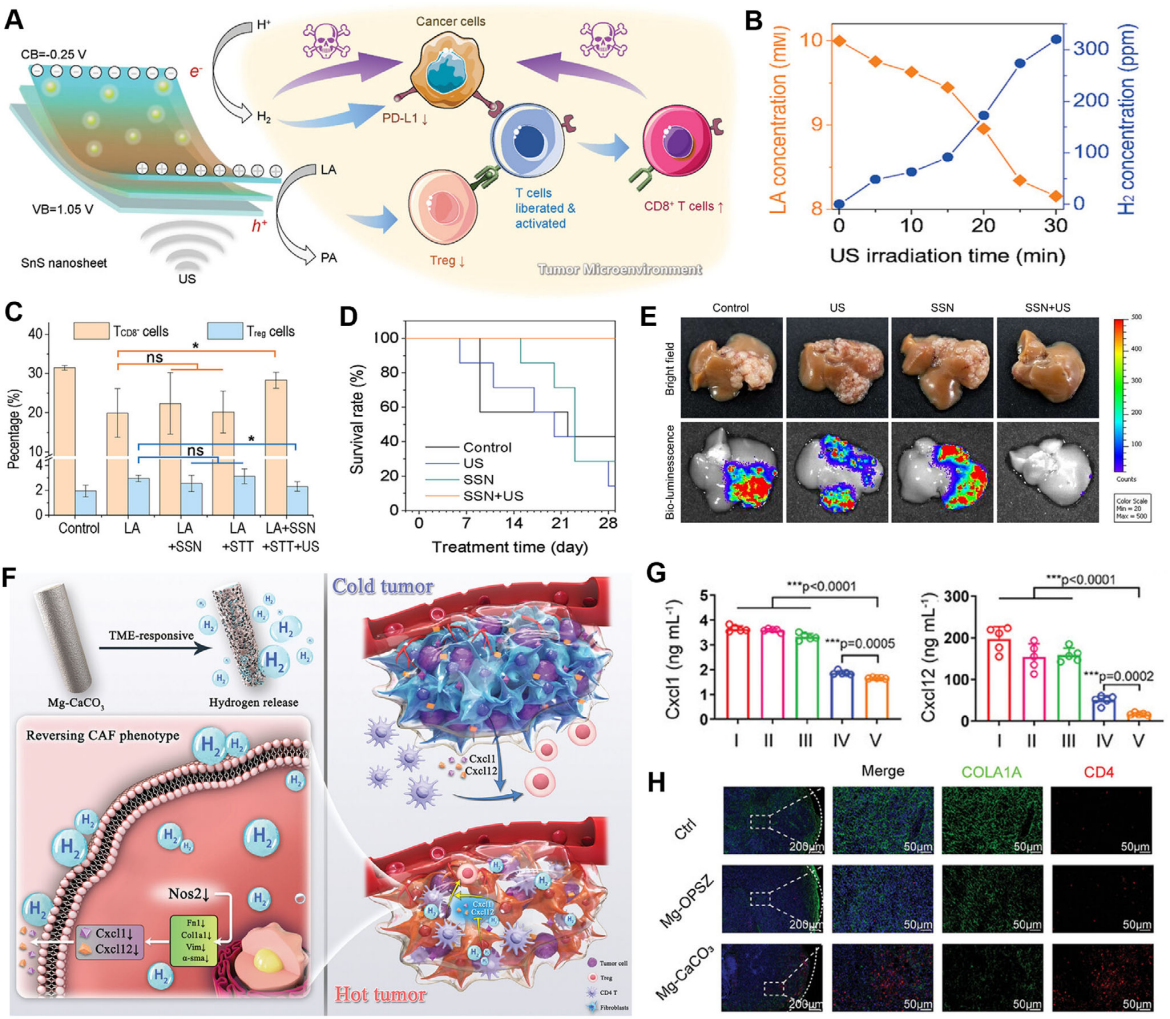
A) Schematic illustration of the mechanism of SnS nanosheets‐mediated piezoelectrocatalytic H_2_ generation and LA deprivation for tumor immunoactivation. B) The intracellular SSN‐mediated piezoelectrocatalytic H_2_ generation and LA consumption. C) The comparison of the proportion of T_CD8_^+^ and T_reg_ cells among various treatments. D) The survival rate of liver tumor‐bearing mice after treatment. E) Representative digital photographs of the dissected tumors after 28 days of treatment. Reproduced with permission.^[^ ^70^ ^]^ Copyright 2023, Wiley‐VCH. F) Schematic illustration of TME remodeling and tumor inhibition mechanisms of Mg‐CaCO_3_. G) Quantification of the concentration of Cxcl1 and Cxcl12 levels in the supernatant of CAFs with different treatments. H) Immunofluorescence images of tumors collected from Balb/c mice after different treatments. Reproduced with permission.^[^ ^75^ ^]^ Copyright 2024, Wiley‐VCH.

Achieving targeted immunotherapy for tumors is crucial for effective and low‐toxicity cancer treatment. Given the significant role of overexpressed lactate (LA) in the immune microenvironment of tumors, Guo et al. employed 2D SnS nanosheets (SSN) in an in situ liver cancer model.^[^ ^70^ ^]^ The piezoelectric catalysis‐induced H_2_ generation and concurrent LA depletion work synergistically to release effector CD^8+^ T (T_CD8+_) cells from immune suppression by tumor cells and inhibiting regulatory T (T_reg_) cells, thereby activating tumor immunity and enabling efficient liver cancer immunotherapy (**Figure** **6**A). As shown in Figure 6B, under ultrasound (US) stimulation, SSN can simultaneously catalyze H_2_ generation and LA consumption within cells. The piezoelectric catalysis‐mediated H_2_ generation by SSN suppresses PD‐L1 overexpression in IFN‐γ‐stimulated Hepa 1–6 cells, offering the potential for tumor immune activation by targeting the PD‐L1/PD‐1 pathway. On one hand, the H_2_ generated by piezoelectric catalysis inhibits the expression of PD‐L1 on tumor cells, thus releasing immune‐suppressed T_CD8+_ cells. On the other hand, LA depletion via piezoelectric catalysis inhibits Treg cells and activates T_CD8+_ cells (Figure 6C). Throughout the 28‐day treatment period, the survival rate of treated mice remained at 100% (Figure 6D). At the end of the treatment, bioluminescence imaging of the extracted liver tissues showed that the US‐driven SSN+US piezoelectric catalysis therapy nearly completely eradicated the orthotopic liver tumors (Figure 6E).

There has been extensive research on H_2_ delivery strategies targeting cancer cells, but the immunomodulation effect of H_2_ delivery on the TME remains unclear. Cancer‐associated fibroblasts (CAFs) play a crucial role in promoting tumor progression by creating physical barriers to drug delivery and immune cell infiltration, while secreting factors such as TGF‐β, IL‐6, and CCL2 that suppress T cell activity and recruit immune‐suppressive cells.^[^ ^126^, ^127^, ^128^ ^]^ Targeting CAFs has the potential to enhance anti‐tumor immunity and improve therapeutic outcomes. Sun et al. used Mg rods coated with calcium carbonate (Mg‐CaCO_3_) to achieve rapid and stable H_2_ release in the weakly acidic TME (Figure 6F).^[^ ^75^ ^]^ The released H_2_ reduces CAFs' generation of pro‐tumor and immune‐suppressive factors (Cxcl1 and Cxcl12) and boosts CD^4+^ T (T_CD4+_) cell‐mediated anti‐tumor immunity (Figure 6G,H). By reversing the pro‐cancer phenotype of CAFs, this system can convert “cold” tumors into “hot” tumors, ultimately demonstrating enhanced immune activation.

### 5.5 Antibacterial

**Figure 7:**
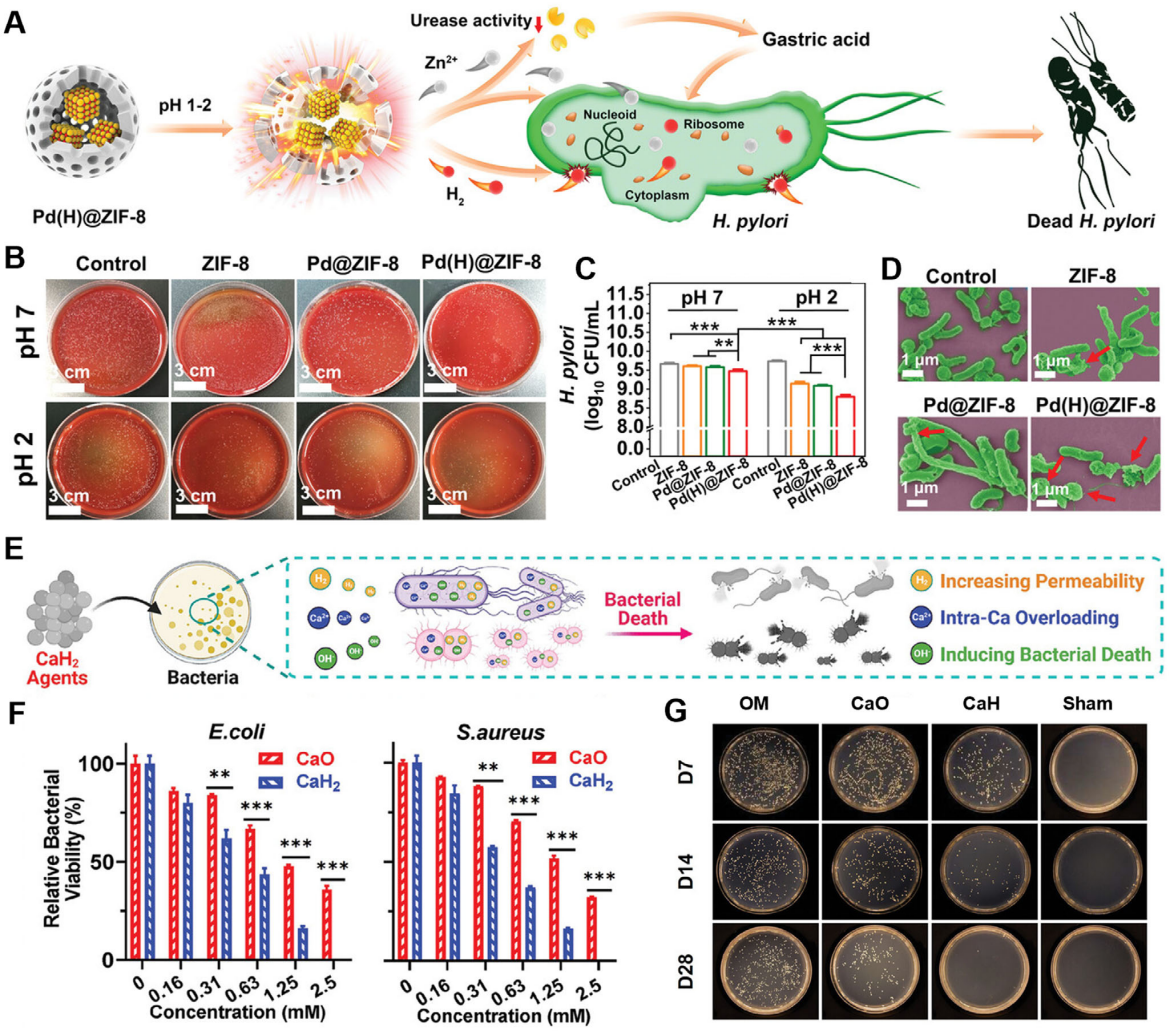
A)The antibacterial mechanism diagram of Pd(H) @ ZIF‐8 under gastric acid condition. B) Images of *H. pylori* colony plates and C) growth of *H. pylori* counted by CFUs per milliliter after treatment with different samples under different pH conditions. D) SEM images of physiological morphological changes of *H. pylori* after different treatments. Reproduced with permission.^[^ ^133^ ^]^ Copyright 2021, Wiley‐VCH. E) Schematic diagram showing the antibacterial performance of CaH_2_ materials. F) Relative percentages of bacteria remaining on *E. coli* and *S. aureus* after treatments. G) Spread‐plate assays of the bacterial content from the bone marrow after treatment. Reproduced with permission.^[^ ^136^ ^]^ Copyright 2024, Wiley‐VCH.

Multidrug‐resistant (MDR) bacterial infections present a major public health challenge, as antibiotic overuse has led to significant resistance, rendering conventional antibacterial therapies increasingly ineffective.^[^ ^129^, ^130^ ^]^ H_2_‐mediated treatment has recently shown promise in anti‐infective applications, particularly in bacterial infection management and immune modulation.^[^ ^131^ ^]^ H_2_ can enhance bacterial membrane permeability to facilitate drug or ion penetration, while also scavenging ROS to alleviate inflammatory responses, offering a safe and versatile approach to infection control.^[^ ^132^ ^]^ For instance, Wang et al. developed a metal‐organic framework‐based H_2_ release platform (Pd(H)@ZIF‐8) that demonstrated efficacy against *Helicobacter pylori* (*H. pylori*) infection.^[^ ^133^ ^]^ In gastric environments, Pd(H)@ZIF‐8 decomposes to release H_2_ along with Zn^2+^ ions (**Figure** **7**A). The H_2_ disrupts bacterial membrane integrity, thereby enhancing Zn^2+^ cellular uptake, leading to intracellular leakage, metabolic disruption, and effective bacterial eradication (Figure 7B–D). Yu et al. further utilized PdH nanohydrides for H_2_‐photothermal synergistic antibacterial therapy, revealing robust antibacterial, anti‐biofilm, and wound‐healing properties.^[^ ^131^ ^]^ Mechanistically, in the oxidative stress pathway, the H_2_‐photothermal synergy upregulates bacterial metabolic genes (*dmpI*, *narJ*, *nark*), increasing the expression of oxidative enzymes, which elevates ROS levels and causes DNA damage. In the membrane damage pathway, this combination severely disrupts bacterial membranes, facilitating H_2_ diffusion into the bacteria and inducing intracellular DNA leakage.

H_2_ has gained considerable attention for its potential in treating deep‐seated bacterial infections, such as sepsis, due to its anti‐inflammatory properties, which contribute to survival benefits and additional biological effects, including anti‐apoptotic, anti‐shock, and autophagy‐regulating activities.^[^ ^134^ ^]^ Controlled delivery and active propulsion of H_2_ offer significant potential for managing these infections. Tu et al. developed Mg‐based micromotors (Mg‐Tob motor) for in vivo combination therapy of sepsis in the cecal ligation and puncture mouse model.^[^ ^76^ ^]^ The Mg‐Tob motor, prepared by asymmetrically coating Mg microparticles with a biodegradable PLGA layer followed by a tobramycin‐loaded alginate‐chitosan hydrogel, utilize peritoneal fluid in septic mice to fuel the Mg‐water reaction. Sustained H_2_ generation at the micromotor interface propels the micromotor and acts as an anti‐inflammatory agent. Concurrently, the continuous H_2_ release enables controlled antibiotic tobramycin delivery, achieving dual immunomodulation and anti‐infective therapeutic effects. Similarly, a self‐propelling Janus gallium /Mg bimetallic micromotor is designed to release H_2_, which helps it breakthrough biological barriers like saliva and gingival crevice fluid to reach the bottom of periodontal pockets, exerting both antibacterial and anti‐inflammatory effects.^[^ ^135^ ^]^ Additionally, CaH_2_ can generate H_2_ to enhance bacterial membrane permeability (by ≈1.5‐fold), facilitating OH⁻ and Ca^2+^ entry into the bacteria (Figure 7E).^[^ ^136^ ^]^ The resulting alkaline environment suppresses bacterial proliferation and induces bacterial death, while H_2_ scavenges excess ROS in infected areas. When applied in a mouse tibial osteomyelitis model infected with *Staphylococcus aureus* (*S. aureus*), CaH_2_ significantly reduced bacterial counts and local inflammation more effectively than calcium oxide (CaO), offering a safer and more effective treatment for deep tissue infections such as osteomyelitis (Figure 7F,G). These studies underscore the multidimensional application of H_2_ in antibacterial therapies. Through innovative H_2_ delivery platforms, precise immune modulation in bacterial infections has been achieved, providing therapeutic options for combating MDR bacterial infections.

### 5.6 Other Inflammatory Disease Regulation

**Figure 8:**
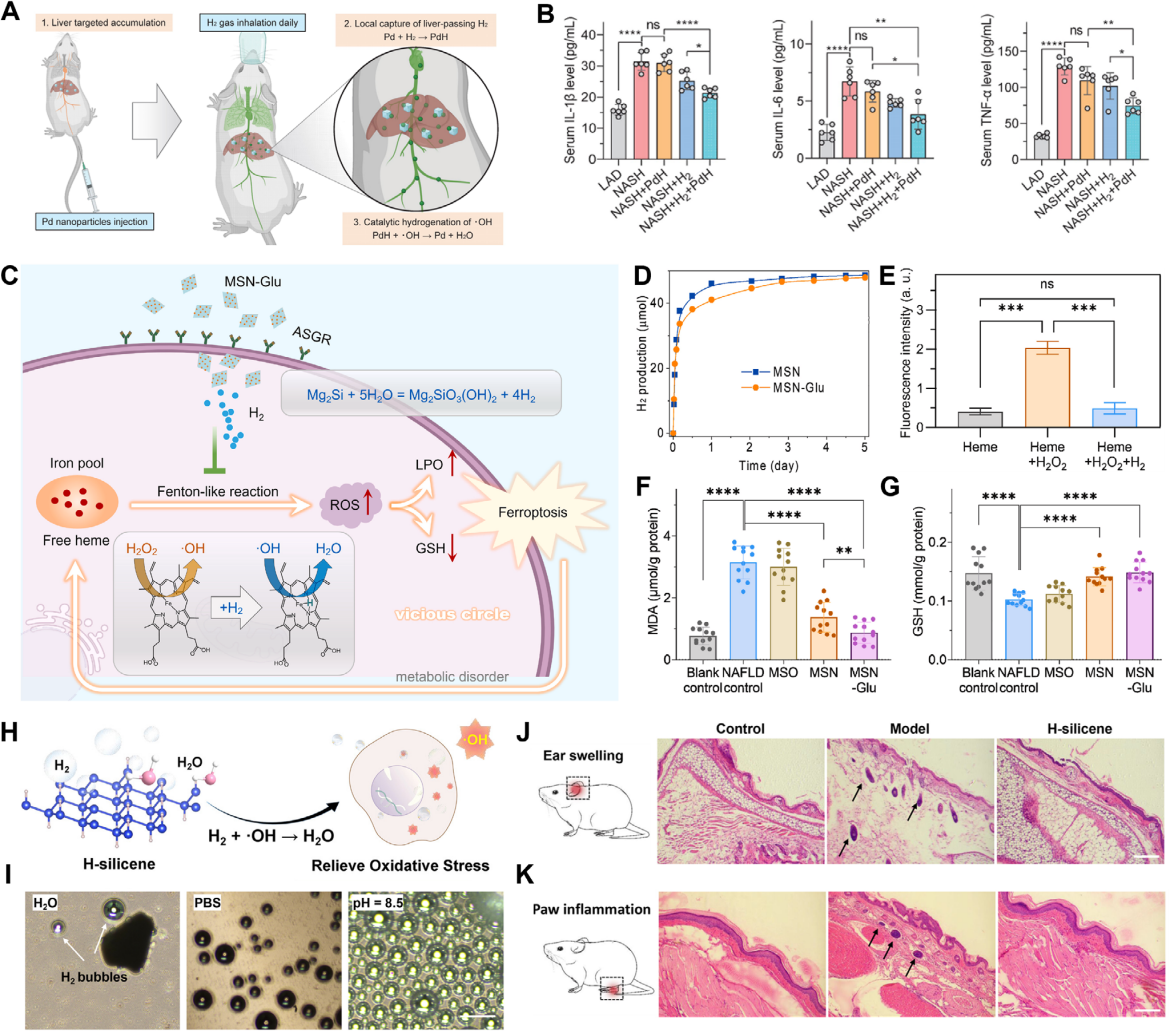
A) Schematic illustration of the therapeutic strategy and mechanism for local H_2_ capture and catalytic ·OH hydrogenation with Pd nanoparticles in liver. B) The levels of serum cytokines including IL‐1β, IL‐6 and TNF‐α in liver sections at the end of treatment. Reproduced with permission.^[^ ^79^ ^]^ Copyright 2023, Ivyspring International Publisher. C) Schematic illustration of the hepatocyte‐targeted delivery of H_2_ by MSN‐Glu for blocking the heme‐catalyzed vicious circle of NAFLD. D) The H_2_ release profiles of MSN and MSN‐Glu. E) Quantitation of relative ·OH levels by measuring the intracellular fluorescence intensity. ns, no significant difference. F,G) Hepatic MDA (F) and GSH (G) contents. Reproduced with permission.^[^ ^78^ ^]^ Copyright 2023, Elsevier. H) Schematic illustration of ROS scavenging in cells under oxidative stress by H_2_ released from H‐silicene. I) Optical microscopic images of H_2_ bubble generation from H‐silicene during reaction with H_2_O, PBS, and NaOH solution (pH = 8.5); scale bar: 200 µm. J) Schematic illustration of the ear swelling model and H&E staining of mouse ears after different treatments; scale bar: 200 µm. K) Schematic illustration of the paw inflammation models and H&E staining of mouse footpads after different treatments; scale bar: 200 µm. Reproduced with permission.^[^ ^84^ ^]^ Copyright 2022, American Chemical Society.

Chronic liver diseases (CLDs), including non‐alcoholic fatty liver disease (NAFLD), are notoriously difficult to treat due to the progressive inflammation, fibrosis, and metabolic complications that lead to cirrhosis or liver cancer.^[^ ^137^, ^138^ ^]^ Current treatments for CLDs, such as anti‐inflammatory drugs, often bring toxic side effects and burden the liver further.^[^ ^139^, ^140^ ^]^ Recent developments in H_2_ delivery materials offer a new therapeutic approach by leveraging H_2_’s broad‐spectrum immunomodulation properties. For instance, Pd nanocrystals have shown promise in liver‐targeted H_2_ delivery. These nanoparticles, administered intravenously, accumulate in the liver and capture H_2_ during gas inhalation, storing it in a solid form as PdH (**Figure** **8**A).^[^ ^79^ ^]^ This allows for the localized catalytic hydrogenation of toxic radicals, such as ·OH, enhancing the effectiveness of H_2_ delivery. The controlled release of H_2_ directly in the liver ensures a significant reduction in pro‐inflammatory cytokine levels, including IL‐1β, IL‐6, and TNF‐α, thereby attenuating inflammation (Figure 8B). The therapeutic efficacy of H_2_ treatment is closely related to its dosage. Due to the inherent diffusion characteristics of H_2_, it cannot accumulate in high concentrations within the liver.^[^ ^141^ ^]^ Therefore, targeting and sustained release of H_2_ to liver cells are crucial for improving therapeutic outcomes, although this remains a challenge. Building on this foundation, He's group has innovatively developed N‐(3‐triethoxysilylpropyl)‐glucosylamide (Glu)‐modified Mg_2_Si nanosheets (MSN‐Glu), which actively target hepatocytes via recognition of asialoglycoprotein receptors on the cell surface, enabling precise H_2_ delivery to liver cells (Figure 8C). The developed MSN‐Glu nanoparticles exhibit a remarkable H_2_ delivery capacity of 105 mg/g, which is 6.6 × 10^4^ times higher than that of saturated hydrogen‐rich water, and sustain H_2_ release for up to 8 days under physiological conditions (Figure 8D). In vitro experiments demonstrated that the delivered H_2_ effectively blocks heme‐based Fenton‐like reactions (Figure 8E). In a NAFLD mouse model, treatment with MSN‐Glu resulted in complete clearance of malondialdehyde (MDA), a sensitive marker of oxidative damage and lipid peroxidation, while restoring glutathione (GSH) levels to normal, indicating a recovery of liver function (Figure 8F,G). This targeted delivery approach not only reduces systemic side effects but also holds substantial potential for improving therapeutic outcomes in the treatment of CLDs.

Acute inflammation is often exacerbated by oxidative stress, where excessive ROS play a central role.^[^ ^142^ ^]^ Recent innovations include the use of ultrathin 2D silicon nanosheets, specifically H‐silicene, which have shown remarkable potential in producing H_2_ on demand.^[^ ^143^ ^]^ Silicon, while abundant and cost‐effective, traditionally has low reaction rates with water. However, nanoscaled silicon exhibits significantly enhanced H_2_ generation due to its high surface area and active sites. The development of H‐silicene, a form of silicon nanosheet with hydrogenated dangling bonds, has addressed this limitation (Figure 8H).^[^ ^84^ ^]^ This material reacts vigorously with water, generating H_2_ in substantial quantities (Figure 8I). The generated H_2_ acts as a potent antioxidant, effectively scavenging ROS and alleviating oxidative stress associated with acute inflammation. In mouse ear swelling and paw inflammation models, H‐silicene has demonstrated excellent anti‐inflammatory effects (Figure 8J,K). The high surface area and active hydrogen bonds facilitate rapid H_2_ release, which in turn protects cells from oxidative damage and apoptosis. This capability makes H‐silicene a viable candidate for acute inflammation management, where immediate and effective reduction of ROS is crucial. Furthermore, the unique properties of H‐silicene extend beyond just H_2_ generation. Its high biocompatibility and non‐toxic byproducts, primarily silica, ensure that it is suitable for therapeutic use. This makes it an attractive option for treating a range of inflammatory conditions where rapid and localized H_2_ delivery can provide significant relief. In summary, the development of advanced H_2_ delivery systems, particularly through the use of 2D silicon nanosheets like H‐silicene, represents a significant advancement in managing acute inflammation. These systems offer a promising approach to delivering H_2_ efficiently and safely, addressing the need for effective immunomodulation in inflammation‐related diseases.

## 6 Conclusion and Prospects

In conclusion, H_2_ delivery offers a promising approach for precision immunomodulation, effectively reducing oxidative stress and modulating inflammatory responses in various diseases. Recent advancements, particularly the identification of Fe‐porphyrin as a biological target of H_2_, have revealed the fundamental reasons behind the therapeutic potential of H_2_ in treating various diseases. This discovery not only deepens our understanding of the biomedical effects of H_2_ but also provides a theoretical foundation for the personalized design of H_2_ delivery systems. By improving targeting accuracy, biosafety, and imaging capabilities, the field is poised to enhance the effectiveness of H_2_ delivery, leading to innovative solutions for currently intractable diseases.

Enabled by the developments in nanotechnology, smart H_2_ delivery systems allow for controlled, targeted, and stimuli‐responsive H_2_ release, offering significant therapeutic potential in regulating immune‐related diseases. Despite these promising advancements, the clinical translation of H_2_ delivery for immunomodulation still faces challenges. Key areas for future research include improving the biosafety of H_2_ delivery materials, optimizing delivery mechanisms for enhanced targeting, and understanding the dose‐dependent therapeutic effects of H_2_ across different disease states. Additionally, integrating real‐time monitoring technologies for precise control of H_2_ release and its therapeutic effects is an area ripe for exploration. Future efforts should also focus on developing subcellular imaging techniques for H_2_ detection and exploring the feasibility of blocking Fenton‐like reactions involving Fe/Mn/Zn porphyrins, which may impact the progression of chronic inflammatory diseases. Moreover, the development of intelligent, multifunctional H_2_‐delivery systems that combine diagnostics, therapy, and real‐time monitoring will pave the way for more effective H_2_‐based treatments in the future. By improving targeting accuracy, biosafety, and imaging capabilities, the field is poised to enhance the effectiveness of H_2_ delivery, leading to innovative solutions for currently intractable diseases.

## Conflict of Interest

The authors declare no conflict of interest.
